# Genome-wide analysis and functional annotation of chromatin-enriched noncoding RNAs in rice during somatic cell regeneration

**DOI:** 10.1186/s13059-022-02608-y

**Published:** 2022-01-19

**Authors:** Yu-Chan Zhang, Yan-Fei Zhou, Yu Cheng, Jia-Hui Huang, Jian-Ping Lian, Lu Yang, Rui-Rui He, Meng-Qi Lei, Yu-Wei Liu, Chao Yuan, Wen-Long Zhao, Shi Xiao, Yue-Qin Chen

**Affiliations:** 1 Guangdong Provincial Key Laboratory of Plant Resources, State Key Laboratory for Biocontrol, School of Life Science, Sun Yat-Sen University, Guangzhou, 510275 People’s Republic of China; 2 MOE Key Laboratory of Gene Function and Regulation, Sun Yat-sen University, Guangzhou, 510275 People’s Republic of China

**Keywords:** Chromatin-enriched noncoding RNAs, Somatic cell regeneration, Rice traits

## Abstract

**Background:**

Plants have the remarkable ability to generate callus, a pluripotent cell mass that acquires competence for subsequent tissue regeneration. Global chromatin remodeling is required for this cell fate transition, but how the process is regulated is not fully understood. Chromatin-enriched noncoding RNAs (cheRNAs) are thought to play important roles in maintaining chromatin state. However, whether cheRNAs participate in somatic cell regeneration in plants has not yet been clarified.

**Results:**

To uncover the characteristics and functions of cheRNAs during somatic cell reprogramming in plants, we systematically investigate cheRNAs during callus induction, proliferation and regeneration in rice. We identify 2284 cheRNAs, most of which are novel long non-coding RNAs or small nucleolar RNAs. These cheRNAs, which are highly conserved across plant species, shuttle between chromatin and the nucleoplasm during somatic cell regeneration. They positively regulate the expression of neighboring genes via specific RNA motifs, which may interact with DNA motifs around cheRNA loci. Large-scale mutant analysis shows that cheRNAs are associated with plant size and seed morphology. Further detailed functional investigation of two che-lncRNAs demonstrates that their loss of function impairs cell dedifferentiation and plant regeneration, highlighting the functions of cheRNAs in regulating the expression of neighboring genes via specific motifs. These findings support *cis*- regulatory roles of cheRNAs in influencing a variety of rice traits.

**Conclusions:**

cheRNAs are a distinct subclass of regulatory non-coding RNAs that are required for somatic cell regeneration and regulate rice traits. Targeting cheRNAs has great potential for crop trait improvement and breeding in future.

**Supplementary Information:**

The online version contains supplementary material available at 10.1186/s13059-022-02608-y.

## Background

Plant development is driven by specific patterns of gene expression that are tightly regulated in a spatio-temporal manner. Chromatin remodeling plays a central role in establishing transcriptional programs required for organ initiation and differentiation [1–3]. Whereas epigenetic states in animals are established early during embryonic development, epigenetic mechanisms in plants also operate during post-embryonic developmental transitions, such as organogenesis and flowering [4, 5]. The chromatin remodeling activities in plants provide a higher degree of flexibility that likely underlies their developmental plasticity. Specifically, multiple detached plant tissues are capable of forming a pluripotent cell mass called callus, which in turn can regenerate into different organs and form a new plant, a process known as somatic embryogenesis [6, 7]. Various genetic and physiological factors trigger somatic embryogenesis in different types of somatic cells. Chromatin remodeling is believed to play a central role during somatic cell reprogramming and pluripotent cell differentiation [6–8].

Recent genomic research has revealed that the genomes of different organisms, including plants, are more prevalently transcribed than previously thought [9, 10]. Mammalian and plant genomes express not only protein-coding mRNAs but also a large repertoire of non-coding RNAs (ncRNAs) with regulatory roles in different layers of gene expression [9, 10]. In mammals, many ncRNAs appear to act directly on chromatin, as exemplified by various long non-coding RNAs (lncRNAs). Some lncRNAs mediate genomic interactions predominantly *in cis*, whereas others are capable of acting extensively *in trans* [11–14]. These findings point to a role for specific RNA–chromatin interactions in regulating gene expression. lncRNA-directed processes also function in dosage compensation in Drosophila, where the localization of the histone acetyltransferase MOF to the male X chromosome is dependent on roX ncRNAs [15]. Moreover, recent findings suggest that ncRNAs are integral components of chromatin [12, 13] that play an important role in the higher-order chromatin structure of pericentric heterochromatin by organizing heterochromatic components [16, 17]. In plants, lncRNAs have been reported to interact with chromatin remodelers [18]. These observations underline the importance of ncRNAs as cofactors in modifying chromatin via the recruitment of chromatin-remodeling complexes. However, the identities of chromatin-enriched ncRNAs (cheRNAs) in plants have not yet been addressed on a global scale.

In this study, we asked whether specific ncRNA–chromatin interactions participate in regulating gene expression during somatic cell regeneration in plants and, if so, what the identities of these chromatin-interacting ncRNAs are. Specifically, during somatic cell reprogramming and pluripotent cell differentiation, what is the landscape of chromatin-associated ncRNAs? Understanding the composition, characteristics, and functions of cheRNAs during cellular reprogramming would provide insight into the molecular network regulating cell pluripotency, thereby facilitating crop breeding.

To address these issues, we used in vitro–cultured embryogenic rice callus as a model to investigate chromatin-interacting ncRNAs associated with embryogenesis and post-embryonic development. Embryogenic calli derived from mature embryos contain a set of homogeneous pluripotent cells that are thought to represent proliferating meristematic tissues. When cultured in the appropriate medium, these embryogenic calli undergo somatic embryogenesis. The cell division, cytodifferentiation, and embryogenesis of embryogenic calli are consistent with these biological processes in vivo. We identified 2284 cheRNAs, including lncRNAs, small nucleolar RNA (snoRNAs), and tRNAs. These cheRNAs are highly conserved and represent subclasses of rice ncRNAs with developmental-stage-specific enrichment patterns. During somatic cell regeneration, cheRNAs shuttle between chromatin and the nucleoplasm, where they regulate the expression of specific protein-coding genes via specific RNA motifs, which might interact with DNA motifs around cheRNA loci. Large-scale analysis of mutant and transgenic rice plants indicated that cheRNAs regulate yield-related traits in rice. Thus, cheRNAs have great potential as targets for trait improvement and crop breeding in the future.

## Results

### Global view of RNA-chromatin interactions during somatic cell regeneration and differentiation in rice

**Fig. 1:**
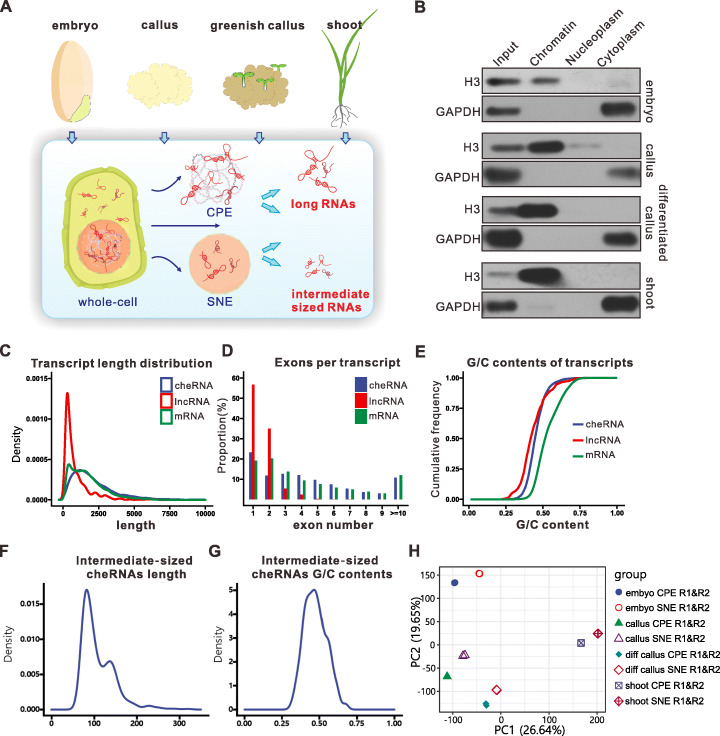
Nuclear fractionation to isolate chromatin-associated RNAs from four tissues and the properties of rice cheRNAs. **A** Depiction of the nuclear fractionation procedure. **B** Immunoblot analysis of histone H3 and GAPDH in chromatin, the nucleoplasm, and the cytoplasmic fraction. **C** Density plot of transcript length distributions for cheRNAs, lncRNAs (annotated using NONCODEv6), and mRNAs (annotated using MSU7.0). **D** Number of exons per transcript for cheRNAs, annotated lncRNAs, and mRNAs. **E** Cumulative distribution of G/C content for cheRNAs, annotated lncRNAs, and mRNAs transcripts. **F** Density plot of transcript length distributions for intermediate-sized cheRNAs (including che-snoRNAs, cheRNA-tRNAs, and che-snRNAs, *n* = 440). **G** Density plot of G/C content for intermediate-sized cheRNA transcripts (including che-snoRNAs, che-tRNAs, and che-snRNAs, *n* = 440). **H** Principal component analysis (PCA) of transcript levels (FPKM) in the CPE and SNE fractions from four tissues with two biological repetitions

To identify regulators of chromatin reprogramming that underlie cell fate changes, we characterized the landscape of chromatin-associated RNAs during callus induction, proliferation, and regeneration. Four different rice tissues were collected for the fractionation of nuclei (Fig. 1A), including (1) mature embryos, (2) undifferentiated embryogenic callus, (3) greenish, partially regenerated (differentiated) callus after over 8 weeks of subculture (every 2 weeks on the same medium), and (4) shoots. We developed a method to separate both nucleoplasmic RNAs and chromatin-associated RNAs. In brief, different rice samples were ground and subjected to cell lysis, and the nucleus fraction was collected. This fraction was further divided into the nucleoplasmic and chromatin fractions, and RNAs were isolated individually from these fractions for sequencing (Additional file 1: Fig. S1A). Stripping highly abundant mRNA from the chromatin pellet with urea was critical for identifying chromatin pellet extract (CPE) transcripts because it effectively magnified the coverage depth of low-abundance RNA species. The fractionation was validated by confirming robust chromatin enrichment of histone H3 and cytoplasm enrichment of GAPDH (Fig. 1B).

We then performed transcriptome sequencing (RNA-seq) and deep sequencing of intermediate-sized RNAs (50 to 300 nt, respectively) of the resulting chromatin pellet extract (CPE), soluble-nuclear extract (SNE), and the input sample, yielding > 2750 million mapped reads (Additional file 1: Fig. S1B; Additional file 2: Table S1). De novo–assembled transcripts from both the CPE and SNE fractions were mapped to 65,703 distinct loci in the annotated rice genome from the Rice Genome Annotation Project (MSU7.0). Each transcript was scored for its abundance in the CPE vs. SNE fraction; transcripts with a relative abundance in CPE versus SNE > 1.2 (adjusted *p* value < 0.05) were defined as chromatin-enriched transcripts. The detected chromatin enriched RNAs include 81.5% mRNAs and 18.5% ncRNAs. The chromatin-enriched ncRNAs have a significantly higher chromatin enrichment ratio than those of total mRNAs and the annotated lncRNAs, and these chromatin-enriched ncRNAs were named cheRNAs and used for the following studies. For better recognizing each XLOC transcript in the genome, these cheRNAs are named according to the type of the cheRNAs and their number coordinating in chromosome (Additional file 1: Fig. S2A; Additional file 3: Table S2).

The coding potential of cheRNAs was much lower than that of protein-coding genes but slightly higher than that of lncRNAs (Additional file 1: Fig. S2B). We statistically characterized the cheRNAs based on their lengths and GC contents. The average exon size and transcript length of long non-coding cheRNAs (che-lncRNAs; ≥ 200 nt) were higher than those of annotated lncRNAs. The number of exons in these cheRNAs was greater than for the annotated lncRNAs and similar to that of mRNAs (Fig. 1C, D; Additional file 1: Fig. S2C). The average GC content of these long non-coding cheRNAs was similar to that of annotated lncRNAs and lower than that of known protein-coding genes (Fig. 1E). For the intermediate-sized non-coding transcripts (≥ 50 nt, ≤ 300 nt), the length distribution map showed two peaks at ~ 85 nt and ~ 140 nt, respectively (Fig. 1F). The GC content ranged from 25 to 75%, with a peak at 50% (Fig. 1G).

**Fig. 2:**
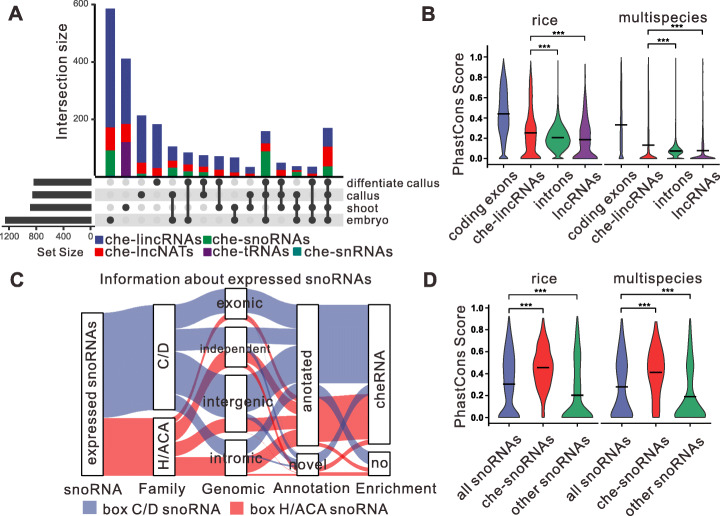
Tissue specificity and conservation of cheRNAs. **A** Upset plot showing the intersections of cheRNAs from mature embryos (*n* = 1245), undifferentiated callus (*n* = 844), differentiated callus (*n* = 828), and shoots (*n* = 876); the bar plot shows the intersection size of each ncRNA category. **B** Violin plot of the distribution of phastCons conservation scores for coding exons and introns of mRNAs (annotated using MSU7.0), che-lincRNAs, and lncRNAs (annotated using NONCODEv6). Mean phastCons scores were derived from 11 way rice whole-genome alignments and multispecies plant samples whole-genome alignments. *p* values were calculated by Wilcoxon Mann-Whitney test, *p* < 0.001 (***). **C** Sankey diagram of expressed snoRNAs data including snoRNA families, genomic organization of the clusters, annotations, and chromatin enrichment. **D** PhastCons conservation score distributions for all snoRNAs, che-snoRNAs, and the remaining unenriched snoRNAs. Mean phastCons scores were derived from 11 way rice whole-genome alignments and 8 way plant whole-genome alignments. *p* values were calculated by Wilcoxon Mann-Whitney test, *p* < 0.001 (***)

We then compared the chromatin-enriched patterns of RNAs during callus induction from embryos, callus differentiation, and plant regeneration. The biological replicates exhibited high reproducibility, and the samples were well separated from each other, suggesting developmental specificity in a substantial fraction of the captured interactions (Fig. 1H). We have also performed H3 ChRIP (Chromatin RNA Immunoprecipitation) to validate the chromatin enrichment of 8 cheRNAs. The results showed that the examined cheRNAs which are chromatin enrichment in callus (*OsCHELIN1575, OsCHELIN2168*, *OsCHENAT0124*, and *OsCHENAT2171*) or in shoot (*OsCHENAT0592*, *OsCHELIN0038*, *OsCHELIN0123*, and *OsCHELIN0456*) were associated to H3 , whereas the lncRNAs identified in nucleoplasm were not associated to H3, which further confirmed their chromatin-bound (Additional file 1: Fig. S2D, E). More cheRNAs were detected in embryos than in the other samples (Fig. 2A). Of the 2284 cheRNAs detected in our experiment, 170 (7.4%) were enriched on chromatin at all developmental stages, while 61.1% of the cheRNAs showed stage-specific enrichment patterns (Fig. 2A). Moreover, 31.5% of these cheRNAs shuttled from the CPE to SNE fragments during somatic cell regeneration and differentiation. Of these shuttled cheRNAs, 773 shuttled from CPE to SNE during callus induction, and 391 shuttled from CPE to SNE during callus differentiation. These results suggest that cheRNAs are under controlled during somatic cell reprogramming.

### Chromatin-interacting ncRNAs are a distinct subclass of ncRNAs

We classified the cheRNAs based on rRNA and tRNA gene annotations from RAP-DB (https://rapdb.dna.affrc.go.jp/), and snRNA and snoRNA gene annotations from the Rfamv14.5 database (http://rfam.xfam.org/). Novel snoRNAs were predicted by snoSeekerNGS and filtered based on the presence of conserved box motifs and expression levels. We identified the che-lncRNAs by comparing each genomic coordinate and strand with MSU7.0 (http://rice.uga.edu/)-annotated mRNA transcripts and estimating the coding potential using the CPC2 program [19]. The chromatin-enriched ncRNAs included lncRNAs (80.74%), snoRNAs (13.75%), tRNAs (5.3%), and snRNAs (0.22%) (Fig. 2A; Additional file 3: Table S2).

Of these cheRNAs, 1527 lacked ncRNA annotations, which was greater than the number of unannotated transcripts (901) enriched in the SNE fraction. Of the lncRNAs, which include both long intergenic noncoding RNAs (lincRNAs) and long noncoding natural antisense transcripts (lncNATs), 77.49% (1429) were not annotated in the PLncDB [20], RNAcentral [21], EVLncRNAs [22], and NONCODE (http://www.noncode.org/index.php) databases and were therefore considered to be novel lncRNAs. These data suggest that, although many studies have identified lncRNAs from rice tissues, most cheRNAs escaped detection using conventional sequencing methods, possibly due to their low abundance and specific subcellular localization. Indeed, only 27.4% of che-lincRNAs are located within 500 bp away from coding genes. Che-lincRNAs exhibited specific strand bias from their putative transcription start sites (TSSs), and the H3K4me3, H3K27ac, and H4K12ac (Additional file 4: Table S3) which were reported to be enriched at gene TSSs showed enrichment at the TSSs of cheRNAs [12, 23] (Additional file 1: Fig. S2F, G). These results suggest that they might not be the byproducts of read-through transcription from upstream genes [12]. One thousand one hundred seventeen of the che-lincRNAs contain repeat elements and might be TE-derived che-lincRNAs. Higher suppressive histone marks (H3K27me3 and H3K9me2) and lower active histone marks (H3K27ac and H3K4me3) were observed at the TE-derived che-lincRNAs loci compared with other che-lincRNAs (Additional file 1: Fig. S2H; Additional file 3: Table S2).

As somatic cell regeneration might be controlled by conserved mechanisms, we investigated whether the che-lincRNAs were more conserved than the annotated lncRNAs. Our analysis showed that the level of conservation of the exons of che-lincRNAs in different plant species is similar to that of coding exons. The level of conservation is relatively modest compared to that of coding exons in different rice varieties, but greater than that of introns and rice lncRNAs (Fig. 2B), suggesting that cheRNAs might be subject to stronger evolutionary pressure than previously characterized lncRNAs.

We also observed many snoRNA-like transcripts on chromatin, including 201 C/D box and 113 H/ACA box snoRNAs. Similarly, chromatin-associated snoRNAs were also identified in mammals [24–26] (Fig. 2C). The 85-nt peak on the length distribution map of intermediate-sized cheRNAs represents box C/D snoRNAs, and the 145-nt peak represents box H/ACA snoRNAs (Fig. 1F; Additional file 1: Fig. S2I). In total, we identified 367 expressed snoRNAs in our dataset. A comparison of these snoRNA-like transcripts with previously annotated snoRNAs in the Rfam database v14.5 and the literature [27] revealed that 49 are completely new snoRNA candidate genes, whereas 35 are chromatin-enriched snoRNAs (Fig. 2C). We analyzed the genomic organization of the novel snoRNAs and found that the majority (277 of 367, 75.5%) of the expressed snoRNAs detected are organized into 96 gene clusters, including 49 intergenic clusters, 35 intronic clusters, and 12 exonic clusters (Fig. 2C), which is in accordance with data previously obtained in rice [27, 28].

We then compared the evolutionary conservation between non-chromatin enriched snoRNAs and che-snoRNAs in different rice varieties, Arabidopsis, Sorghum, and Brachypodium. Similar to che-lincRNAs, che-snoRNAs were significantly more conserved than non-chromatin-enriched snoRNAs (Fig. 2D). When we predicted the potential strong interacted targets of the che-box C/D snoRNAs using PLEXY, 71 of the 237 identified boxC/D snoRNAs had no predicted complementary targets, suggesting that these che-snoRNAs might have different functions from traditional rice snoRNAs.

Collectively, these findings indicate that cheRNAs consist of ncRNAs that previously escaped detection using conventional sequencing methods and could not be annotated. The lengths and GC contents of the cheRNAs are different from those of annotated ncRNAs. In particular, cheRNAs appear to be subject to higher evolutionary pressure than previously characterized lncRNAs and snoRNAs. These characteristics suggest that cheRNAs represent a subclass of rice ncRNAs that might be functional during somatic cell regeneration, an inherent capacity of plants.

### cheRNA dynamics during cellular reprogramming

**Fig. 3:**
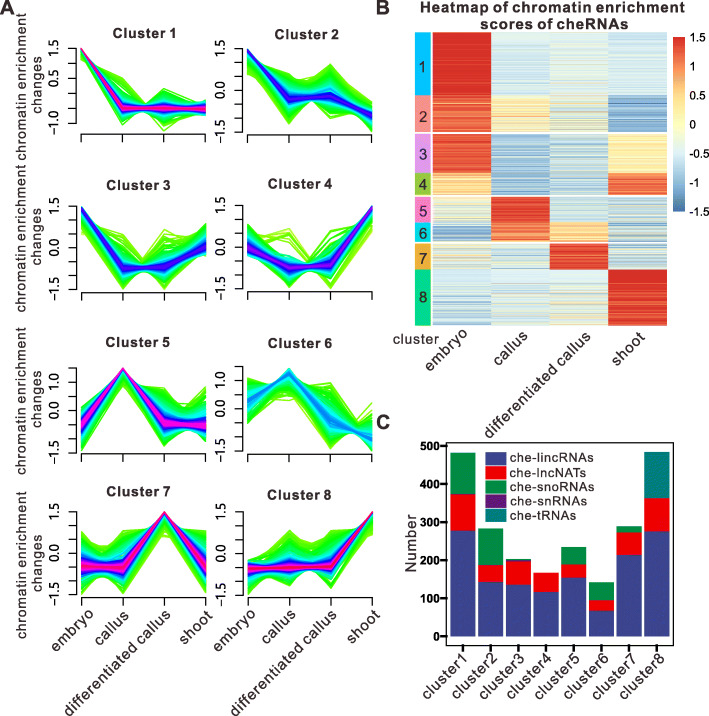
Chromatin enrichment patterns of cheRNAs. **A** Results of time-series clustering analysis by Mfuzz. Purple and blue represent high membership values, and green represents low membership values. The vertical axis represents changes in chromatin enrichment of cheRNAs in each cluster; the horizontal axis represents different tissues (ordered by developmental stage). **B** Heatmap of chromatin enrichment scores of cheRNAs (DESeq2 fold change of CPE versus SNE) in four tissues. **C** Bar plot of the number of ncRNAs in each category (che-lincRNAs, che-lncNATs, che-snoRNAs, che-snRNAs, and che-tRNAs) for each cluster

Having demonstrated that cheRNAs display developmental stage–specific enrichment patterns and may shuttle between chromatin and the nucleoplasm during somatic cell regeneration (Fig. 2A), we next asked how the changes in the chromatin enrichment patterns of cheRNAs are related to cellular reprogramming. To address this issue, we systematically identified the transcriptional shift and chromatin enrichment variation of each cheRNA. A fuzzy *c*-means soft clustering analysis of the cheRNAs grouped them into eight clusters (Fig. 3A, B; Additional file 3: Table S2). For this analysis, we used the scaled chromatin enrichment score (CPE versus SNE fold-changes resulting from differential expression analysis using DESeq2) for calculation with the R package Mfuzz. Only 7.4% of the cheRNAs were associated with chromatin in all four tissues (Fig. 2A, B). The majority shuttled from the CPE to the SNE fraction during somatic cell reprogramming, pointing to their regulatory roles throughout this process (Fig. 2A, B).

When a mature embryo dedifferentiates into pluripotent callus, the closed-chromatin state transforms into an open-chromatin state to allow massive gene reprogramming, conferring various possibilities for differentiation [7]. During this process, there were approximately three times more cheRNAs with declining chromatin enrichment than cheRNAs with increased chromatin enrichment (Fig. 3A, B), implying that more cheRNAs might function in maintaining the differentiated states of somatic cells. During callus differentiation and plant regeneration, only slightly more cheRNAs showed increased chromatin enrichment than declining enrichment (Fig. 3A, B). Thus, cheRNAs consist of both positive and negative regulators of plant regeneration.

Among the eight clusters, the cheRNAs in clusters 1 and 2 were highly chromatin enriched on chromatin in embryos. The cheRNAs in clusters 3 and 4 showed reduced chromatin enrichment during callus induction from embryos and increased chromatin enrichment during plant regeneration, pointing to their roles in plant differentiation. The cheRNAs in clusters 5 and 6 exhibited enrichment patterns opposite to those of cluster 3 and 4 cheRNAs—they were highly enriched on chromatin in callus and showed declining enrichment during differentiation—implying that they might be required for pluripotent cell fate. Cluster 7 and 8 cheRNAs were highly enriched on chromatin in differentiated callus or shoot tissue (Fig. 3A, B). These data indicate that cheRNAs as a group have multiple functions during somatic cell regeneration.

We next analyzed the types of cheRNAs in each cluster. As shown in Fig. 3C, each cluster consists of different types of cheRNAs. che-lncRNAs were distributed in all eight clusters, with a modest preference for clusters 1 (20.6%) and 8 (19.9%). 63.5% of the che-lncRNAs were enriched on chromatin in only a single tissue; whereas 16.6% were enriched on chromatin in three or more tissues, with only the degree of enrichment varying during somatic cell regeneration (Fig. 3C). The chromatin enrichment patterns of the che-snoRNAs were less tissue specific, as 31.2% of che-snoRNAs were enriched on chromatin in only one tissue. Most of these che-snoRNAs are found in clusters 1, 2, and 5 (Fig. 3C). che-tRNAs were only enriched on chromatin in shoots; thus, they are all in cluster 8 (Fig. 3A, C).

Taken together, these results suggest that the cheRNAs are tissue specific during somatic cell reprogramming, and they could shuttle from chromatin to the nucleoplasm. Thus, cheRNAs might function as regulators required for cell dedifferentiation or differentiation.

### Mechanisms underlying the roles of cheRNAs in regulating cellular reprogramming and the expression of differentiation-related genes

**Fig. 4:**
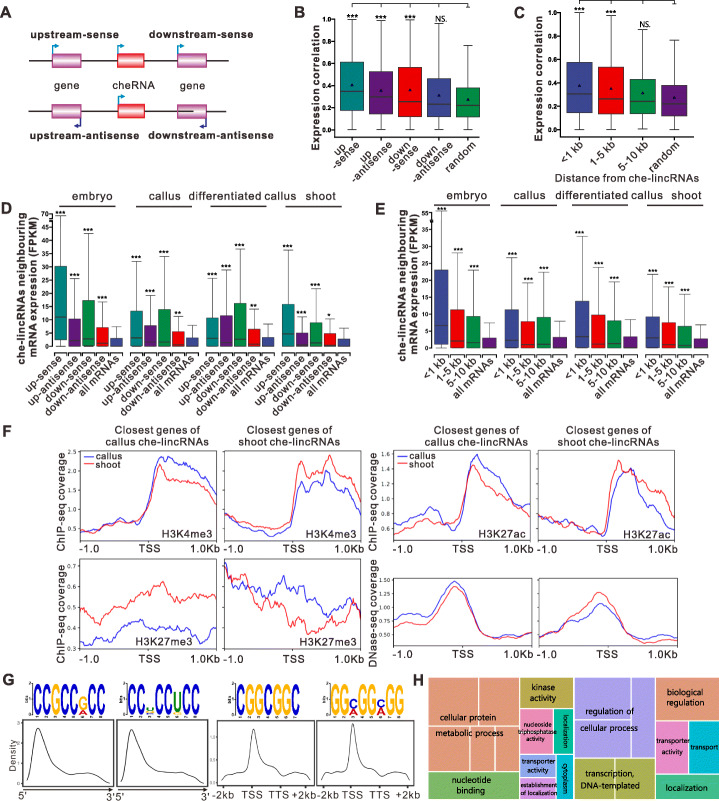
Analysis of the regulatory mechanism of che-lincRNAs. **A** Schematic representation of a cheRNA locus with upstream and downstream coding genes. **B** Comparison of the Pearson correlation coefficients (PCCs) of the absolute values of the expression of che-lincRNAs and their neighboring genes in input, grouped on the basis of strand and orientation to che-lincRNAs. “Random” represents PCC between the expression of che-lincRNAs and randomly selected mRNAs, and triangles represent the mean value of each group. **C** Similar to **B** but grouped by the distance to che-lincRNAs. **D** Comparison of input (total RNAs) expression (FPKM) of the nearest neighboring genes, grouped based on strand and orientation to che-lincRNAs (as indicated in **A**) in four tissues. **E** Similar to **D** but grouped based on the distance to che-lincRNAs. **F** Mean ChIP-seq coverage in callas and shoot of H3K4me3, H3K27ac, H3K27me3, and DNase-seq coverage profiles centered around the TSS of the closest genes of callus-specific or shoot-specific enriched che-lincRNAs respectively (mean CPE FPKM ≥ 5). **G** Consensus motifs in the che-lincRNA transcripts and neighboring gene DNA regions, identified by MEME with default parameters. Relative location was calculated and normalized based on each transcript length, and the density plot was constructed using ggplot2 in R. **H** GO analysis of neighboring genes of che-lincRNAs from the indicated clusters. The significant GO enrichment results (*p* < 0.05) were summarized using REVIGO. The aggregate size indicates the significance levels of the GO term, as determined using the Yekutieli test with false discovery rate correction. In **B–E**, *p* values were calculated by Wilcoxon Mann-Whitney test, *p* < 0.05 (*), *p* < 0.01 (**), *p* < 0.001 (***)

We analyzed the potential mechanisms of cheRNA function during cellular reprogramming and differentiation. Previous studies suggested that cheRNAs might act *in cis* or *in trans* to regulate gene expression [29]. Numerous che-lincRNAs might regulate the expression of their neighboring protein-coding genes [12]. To investigate the *cis*-regulatory activities of che-lincRNAs, we compared the expression patterns of che-lincRNAs and their neighboring genes based on total RNAs.

che-lincRNAs were divided by strand sense and orientation relative to their nearest coding genes (Fig. 4A), and their distribution showed no significant bias. However, che-lincRNA expression levels were more highly correlated with those of nearby protein-coding genes than with those of randomly chosen genes, and che-lincRNAs downstream of their neighbors displayed even stronger expression correlation than che-lincRNAs from other orientation (Fig. 4B; Additional file 5: Table S4). By contrast, the expression levels of che-lncNATs were more highly correlated with the expression levels of neighboring genes on the antisense strands (Additional file 1: Fig. S3). The correlation between the expression levels of che-lincRNAs and their neighboring genes gradually decreased with increasing distance from the che-lincRNAs (Fig. 4C), suggesting that che-lincRNAs might function as local enhancers that affect the expression of multiple genes. We also compared the che-lincRNAs with previously identified enhancers in rice. Fifty-two che-lincRNAs overlapped with enhancers identified by STARR-seq [30], and 274 overlapped with enhancers predicted by DHS [31] (Additional file 1: Fig. S4A), suggesting at least part of them might be *cis*-regulatory elements that function during cellular reprogramming.

Thus, we further analyzed the expression levels of the neighboring genes of che-lncRNAs to investigate the roles of che-lncRNAs in regulating gene expression. While the che-lncNATs did not significantly promote the expression of their neighboring genes (Additional file 1: Fig. S3), a higher correlation with che-lincRNAs tended to result in higher expression of neighboring protein-coding genes (Fig. 4D, E). The effect of che-lincRNAs in promoting neighboring gene expression was significantly higher than those of SNE lincRNAs, annotated lincRNAs, and lncNATs (Additional file 1: Fig. S4B). It has been reported that lncRNAs could regulate gene expression by mediating post-translational modification of histones [18]. Thus, we further analyzed the relationship between che-lincRNAs and the epigenetic activities by using the published data on DNA methylation and histone modification sequencing. The results showed that che-lincRNAs are positively correlated with the H3K4me3 and H3K2ac modifications and chromatin accessibility and negatively correlated with the H3K27me3 modification of their neighboring genes, whereas other histone modifications and DNA methylations are not affected by che-lincRNAs (Fig. 4F; Additional file 1: Fig. S4C, D). lncRNAs undergo sequence-specific interactions with DNA via triple helix (triplex) formation both *in cis* and *in trans*, which allows them to recruit protein complexes to specific genomic regions and regulate gene expression. To analyze whether che-lincRNAs could bind to the DNA regions around the neighboring genes or at *trans* genomic loci, we looked for potential triplexes between che-lincRNAs and the DNA regions of the gene bodies and the regions 1000 bp upstream or downstream of their neighboring genes or across the genome using Triplex Domain Finder (TDF) or Triplexator analysis. This indicated that 390 che-lincRNAs have predicted binding sites on the DNA regions around their neighboring genes (TDF *p* value < 0.05). In addition, che-lincRNAs also have predicted *trans* binding sites across the genome, which inclined to around the TSS of coding genes (Additional file 1: Fig. S4E; Additional file 6: Table S5), pointing to their *in trans* functions. Our data indicate that che-lincRNAs might promote gene expression *in cis*, hinting that che-lincRNAs may have a role in regulating the expression of reprogramming-specific genes rather than genes with basal functions.

We then looked for potential functional motifs in the che-lincRNAs, neighboring genes of the che-lincRNAs, and the genes predicted to form triplexes with che-lincRNAs. Two short motifs, “CCGCCWCC” (H = A or G) and “CCWCCMCC” (W = U or G or C, M = U or G), were identified in 294 and 461 che-lincRNAs, respectively (Fig. 4G). These motifs were also enriched in che-lincRNAs with predicted DNA-binding sites. The motifs were mainly present at the 5′-ends of che-lincRNAs (Fig. 4G). lncRNAs form DNA-RNA hybrids via complementary base pairing [32]. We also identified two short motifs, “CGGCGGC” and “GGNGGNGG” (N = C or A), that were mainly present around the transcription start sites of 619 and 661 neighboring genes of che-lincRNAs, respectively, and genes that were predicted to form triplexes with che-lincRNAs; these sequences are complementary to the motifs enriched in che-lincRNAs (Fig. 4G). In addition, these two motifs share sequence similarity with the reported DNA-binding motifs of lncRNAs in animals [33], pointing to a conserved regulatory mechanism in both plants and animals.

We then examined the coding genes proximal to che-lincRNAs that might be regulated *in cis* by che-lincRNAs in clusters 1 and 2, 3 and 4, 5 and 6, or 7 and 8. The proximal coding genes of che-lincRNAs from different clusters were enriched in different biological processes and functions (Fig. 4H; Additional file 1: Fig. S4F; Additional file 7: Table S6). Cell dedifferentiation and reprogramming lead to comprehensive transcriptional changes [7, 8]. che-lincRNAs in clusters 5 and 6 tended to be chromatin enriched during callus induction and distributed in the SNE fraction during plant regeneration. The proximal coding genes of che-lincRNAs in clusters 5 and 6 primarily included genes encoding proteins required for gene transcription (Fig. 4H; Additional file 7: Table S6). Besides transcriptional regulation, protein phosphorylation is another key factor in plant regeneration, which is essential for hormone signaling pathways. che-lincRNAs in clusters 3 and 4 showed opposite chromatin enrichment patterns from those in clusters 5 and 6. The proximal coding genes of che-lincRNAs in cluster 3 and 4 che-lincRNAs are mainly involved in protein phosphorylation; 15% encode kinases (Fig. 4H; Additional file 7: Table S6). Thus, our data suggest that che-lincRNAs are positive regulators of genes related to somatic cell reprogramming. Collectively, these results suggest that che-lincRNAs might function *in cis* or *in trans* to regulate the expression of specific groups of genes via complementary sequences shared with their target genes.

### cheRNAs are associated with crop traits

The results described above suggest that cheRNAs might regulate somatic cell reprogramming. As extensive regeneration ability is required by plants to ensure their postembryonic development and survival, we investigated the possible roles of cheRNAs in crop development in vivo. Nine public rice mutant databases are currently available (affjp [34, 35], cirad [36, 37], gsnu, ostid [38], pfg [39], rmd [40], ship, trim, and ucd databases), three of which (affjp, cirad, and trim) describe the phenotypes of each mutant. The annotated phenotypes cover all stages of rice development. We performed a preliminary functional analysis of all the identified che-lincRNAs and che-snoRNAs using the nine rice mutant databases. che-lncNATs were not selected for mutant analysis because they partially overlap with protein-coding genes, which might produce false positive results. Our strategy was to perform BLAST analysis of the flanking sequence tags (FSTs) included in each mutant database against the che-lincRNAs and che-snoRNAs and their 1-kb upstream regions (potential promoter regions) separately. A total of 531 cheRNAs were represented by insertional mutants in these databases; these mutants could contribute to the functional analysis of individual cheRNAs.

**Fig. 5:**
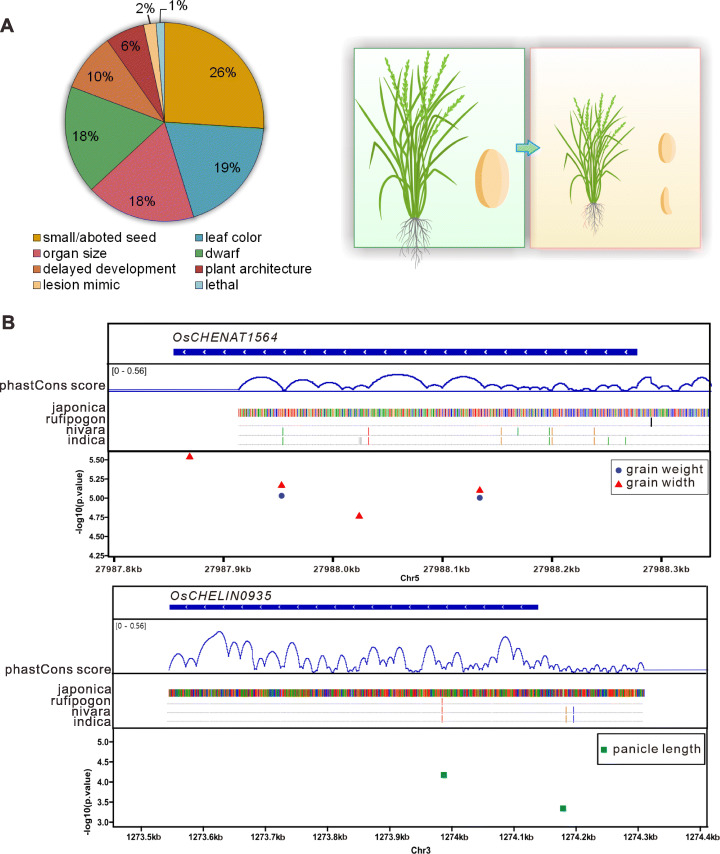
Phenotypic analysis of cheRNAs. **A** The percentages of phenotypes associated with the cheRNA insertion mutants. The organ size and small/aborted seed phenotypes are represented in the schematic diagram. **B** Examples of association analysis of che-lincRNAs OsCHENAT1564 and OsCHELIN0935 with various agricultural traits. The rice species conservation phastCons scores and their 11 way whole-genome alignments were visualized in IGV. The Manhattan plots show SNPs around che-lincRNA genomic regions that are significantly associated with various traits

Annotated phenotypic data were available for the insertional mutants of 206 cheRNAs. We summarized the phenotypes of these mutants and performed statistical analysis. The most frequently occurring phenotypes were small/aborted seeds (26%), leaf color (19%), and altered organ size (18%) (Fig. 5A; Additional file 1: Fig. S4G; Additional file 8: Table S7). These results indicate that cheRNAs have important functions in determining organ size, especially seed size, as well as plant metabolism or plastid development. Notably, these traits directly affect crop yield.

Next, we investigated the single-nucleotide polymorphisms (SNPs) in the cheRNAs to identify trait-associated SNPs in a wide range of rice varieties from the 3K Rice Genomes Project [41, 42]. We identified 343 SNPs in 62 cheRNAs that are significantly associated with grain or panicle traits. For example, che-lncNAT *OsCHENAT1564* contains three SNPs which were significantly associated with grain and panicle size (Fig. 5B). One of these SNPs differentiated during rice domestication. In indica varieties and *Oryza nivara*, the SNP is a A allele, whereas in japonica varieties and *Oryza rufipogon*, it is a G allele (Fig. 5B). Importantly, this SNP was significantly associated with grain width and weight (Fig. 5B). Similarly, a SNP associated with panicle length was identified in the conserved region of che-lincRNA *OsCHELIN0935* (Fig. 5B). These data further emphasize the importance of cheRNAs for rice development and their potential practical value.

### Loss of function of che-lincRNAs impairs cell dedifferentiation and plant regeneration ability

**Fig. 6:**
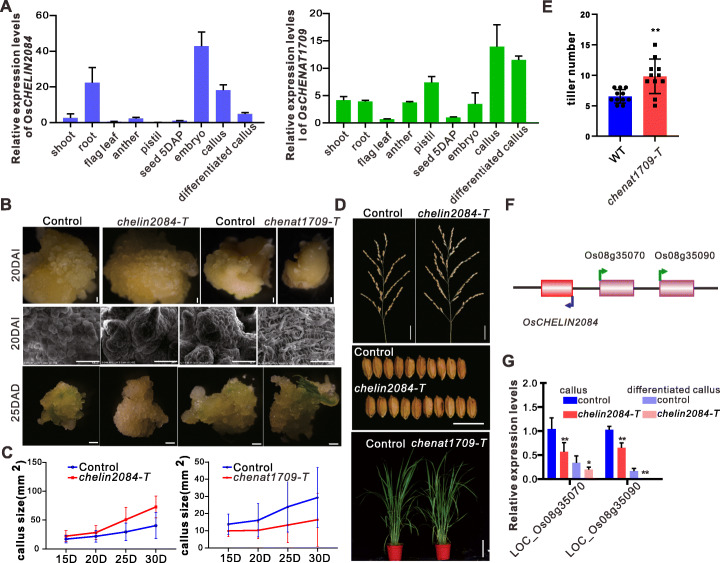
Functional analysis of two cheRNAs. **A** Expression patterns of *OsCHELIN2084* (left) and *OsCHENAT1709* (right). Values are the means ± SD (*n* = 3 replicates, normalized against *ACTIN2*). **B** Callus at 20 days after induction (DAI) and at 25 days after differentiation (DAD) from DJ-WT, *chelin2084-T*, ZH11-WT, and *chenat1709-T* from left to right. Scale bars, 1 mm. **C** Callus size analysis during callus induction. Values are the means ± SD (*n* = 45 calli). **D** Panicles (top) and grains (middle) of DJ-WT and *chelin2084-T*, and whole ZH11 and *chenat1709-T* plants (bottom). Scale bars, 3 cm for panicles, 1 cm for grains and 15 cm for plants. **E** Statistical analysis of tiller number. Values are the means ± SD (*n* = 11 plants). **F** Schematic diagram of the relative locations of *OsCHELIN2084* and its neighboring genes LOC_Os08g35070 and LOC_Os08g35090. **G** Relative expression levels of LOC_Os08g35070, and LOC_Os08g35090 in WT and *chelin2084-T* callus and differentiated callus. Values are the means ± SD (*n* = 3 replicates, normalized against *ACTIN2*). In **E** and **G**, significant differences were identified at 5% (*) and 1% (**) probability levels using two-tailed paired *t*-test

To examine the functions of the cheRNAs, we further analyzed two che-lncRNAs (che-lincRNA *OsCHELIN2084* and che-lncNAT *OsCHENAT1709*) with no annotated phenotypes and compared the phenotypes of their T-DNA insertion mutants and RNAi transgenic plants (Additional file 1: Fig. S5A). *OsCHENAT1709* is highly expressed in callus but expressed at lower levels in differentiated tissues, whereas *OsCHELIN2084* is highly expressed in embryos but more weakly expressed in callus (Fig. 6A). We used T_2_ seeds of loss-of-function T-DNA insertion transgenic plants and RNAi transgenic plants of *OsCHENAT1709* and loss-of-function T-DNA insertion transgenic plants of *OsCHELIN2084* for phenotypic analysis (Additional file 1: Fig. S5A). The three stages that are typically observed during early callus differentiation are the formation of calli, green spots, and shoot primordia. We observed these three stages in the two insertional mutants.

Notably, the calli that were regenerated from the *OsCHELIN2084* loss-of-function mutant (*chelin2084-T*) and the *OsCHENAT1709* loss-of-function and RNAi mutant (*chenat1709-T* and *chenat1709-RNAi*) had opposite phenotypes (Fig. 6B; Additional file 1: Fig. S5B, C, D). When callus formation was induced from embryos, callus formation from *chenat1709-T* and *chenat1709-RNAi* was restrained, whereas callus proliferation from *chelin2084-T* was more rapid than that of wild-type (WT) plants (Fig. 6B; Additional file 1: Fig. S5B - E). After 30 days of induction, the average callus size was 40.41 mm^2^ for WT Dongjin, 72.80 mm^2^ for *chelin2084-T*, and 29.37 mm^2^ for WT Zhonghua11, but only 16.42 mm^2^ for *chenat1709-T* (Fig. 6B, upper panel; Fig. 6C). We have also transfected calli with the RNAi vector of *OsCHELIN2084* and analyzed the phenotypes. The calli transfected with *chelin2084-RNAi* vectors showed rapid proliferation, which is similar with that of the T-DNA insertion mutant of *OsCHELIN2084* (Additional file 1: Fig. S5F, G). Scanning electron microscopy (SEM) revealed that *chelin2084-T* calli consisted of many globular nodules with turgid cells, whereas WT Dongjin and Zhonghua11 calli contained fewer globular nodules and the cells were flaccid, and *chenat1709-T* cells were extremely enlarged (Fig. 6B, middle panel). *chelin2084-T* callus cells were globular and compact, which is characteristic of embryogenic calli [43, 44], whereas *chenat1709-T* callus cells showed the characteristics of non-embryogenic callus [44] (Fig. 6B, upper panel). These results indicate that che-lncNAT *OsCHENAT1709* is required for cell pluripotency, whereas che-lincRNA *OsCHELIN2084* suppresses cell dedifferentiation.

Next, we examined the regeneration process of the mutants. Green spots emerged much earlier in *chenat1709-T* than that of WT plants, whereas *chelin2084-T* showed opposite, the green spots in *chelin2084-T* formed later than that of WT plants (Fig. 6B; Additional file 1: Fig. S5H). Moreover, the green spots of *chenat1709-T* appeared lustrous and were covered with sickle-shaped trichomes, which could eventually transform into shoots, while the green spots of *chelin2084-T* were unorganized, pale green, and covered with white hairs that did not give rise to shoots (Fig. 6B, bottom panel). These results demonstrate that che-lncNAT *OsCHENAT1709* suppresses plant regeneration while che-lincRNA *OsCHELIN2084* is required for regeneration; this is consistent with the expression patterns of these cheRNAs (Fig. 6A). Specifically, *OsCHENAT1709* was highly expressed in callus, and loss of function of this cheRNA impaired cellular pluripotency, whereas *OsCHELIN2084* was highly expressed in embryos, and its loss of function reduced the likelihood of plant regeneration. In addition, we observed significant differences in plant architecture between both the mutants and WT plants. The *OsCHELIN2084* mutant plants (*chelin2084-T*) had longer panicles and wider seeds than the WT (Fig. 6D; Additional file 1: Fig. S5I), while the *OsCHENAT1709* mutant plants (*chenat1709-T* and *OsCHENAT1709*-*RNAi*) had more tillers (Fig. 6 E; Additional file 1: Fig. S5J), suggesting that this cheRNA might regulate cell division or bud formation and development. These findings further support the roles of these cheRNAs in both cellular reprogramming and crop traits.

Together, our findings demonstrate that *OsCHENAT1709* and *OsCHELIN2084* are associated with the process of somatic embryogenesis, in which embryogenic-competent cells respond to environmental and phytohormone signals in culture medium and develop into somatic embryos. These cheRNAs also regulate grain size and panicle size.

Finally, to investigate the potential mechanisms employed by these two cheRNAs in regulating somatic embryogenesis, we examined their neighboring genes. Notably, we detected the “CCGCCWCC” and “CCWCCMCC” motifs in che-lincRNA *OsCHELIN2084* and the “CGGCGGC” and “GGNGGNGG” motifs in the promoter regions of its neighboring genes, encoding Ubiquitin-protein ligase (LOC_Os08g35070) and Subtilisin-like serine protease (LOC_Os08g35090) (Fig. 6F; Additional file 1: Fig. S6A), and their family members have been reported to regulate organ development [45–47]. No che-RNA-associated motif was detected in che-lncNAT *OsCHENAT1709*. Thus, we analyzed the expression correlation between *OsCHELIN2084* and its neighboring genes LOC_Os08g35070 and LOC_Os08g35090, and we found that their expression patterns were positively correlated (Fig. 6G). We further analyzed the phenotypes of the knockout transgenic plants of LOC_Os08g35070 and LOC_Os08g35090 respectively and found that the loss of function of LOC_Os08g35090 showed more rapid callus proliferation and wider seeds than that of the control plants transferred with empty vector (Additional file 1: Fig. S6B, C, D), which is similar with that of the loss of function mutant of *OsCHELIN2084*, while loss of function of LOC_Os08g35070 promoted callus proliferation but not significantly affected seed size (Additional file 1: Fig. S6B, C, D). These results are consistent with the roles of che-lincRNAs in promoting the expression of their neighboring genes, further supporting the hypothesis that che-lncRNAs function as *cis*-regulatory elements during cellular reprogramming.

Collectively, these data suggest that che-lncRNA loci act as transcriptional regulators *in cis* and are required for embryo regeneration.

## Discussion

Plants have the remarkable ability to generate a pluripotent cell mass that acquires competence for subsequent tissue regeneration [48, 49]. This cell fate transition is accompanied by epigenetic changes [6]. Global reprogramming of DNA methylation, histone modification, and chromatin remodeling is required for the cell fate transition [7, 50–52]. Therefore, global changes in the chromatin landscape define gene expression patterns. For example, during callus formation, the loss of DNA methylation deregulates the expression of protein-coding genes involved in certain biological processes. How the global reprogramming of chromatin is regulated during the cell fate transition is not fully understood. In animals, cell totipotency is thought to rely primarily on the unique chromatin of totipotent cells or on an RNA-centric posttranscriptional regulation program [53]. cheRNAs are thought to function as epigenetic regulators that play important roles in creating and/or maintaining chromatin states that influence changes in gene expression during development [11–14]. In this study, we identified cheRNAs from mature rice embryos, callus induced from mature embryos, regenerated greenish calli, and shoots and showed that the cheRNAs likely regulate the expression patterns of specific genes during somatic cell regeneration.

A total of 2284 cheRNAs were identified, which mainly consisted of lncRNAs and snoRNAs. The composition of rice cheRNAs is similar to that reported in mammals, indicating that the roles of lncRNAs and snoRNAs in regulating chromatin status are conserved between plants and animals. In addition, these cheRNAs have different characteristics from other ncRNAs, especially their level of conservation: che-lincRNAs and che-snoRNAs are highly conserved across plant species and in different rice varieties, pointing to the strong evolutionary pressure on cheRNAs and their fundamental functions. For example, in the che-lncNAT *OsCHENAT1564* and che-lincRNA OsCHELIN0935, several SNPs are significantly associated with rice traits. Thus, it is important to further analyze the functions of individual cheRNAs.

Another characteristic of cheRNAs is that their enrichment on chromatin is dynamic during cellular reprogramming, suggesting that they might shuttle between chromatin and the nucleoplasm. Their dynamic chromatin enrichment patterns might be associated with their roles in regulating gene expression during the cell fate transition. For example, chromatin associated lncRNA *XIST in cis* regulates X chromosome inactivity over long genomic distance; and chromatin associated lncRNA *FIRRE* has both *trans*- and *cis*-acting effects on epigenetic features [54]. In addition, the dissociation of lncRNAs from chromatin is also important for their regulatory roles, such as lncRNA *A-ROD* was shown to enhance its upstream gene *DKK1* transcription at its release from chromatin [55]. We have observed correlations between the expression patterns of che-lincRNAs and their adjacent genes, and examined the specific functions of these adjacent genes. We found, for example, that the adjacent genes of che-lincRNAs that are enriched on chromatin during cell dedifferentiation but dissociate from chromatin during plant regeneration primarily include genes encoding proteins required for gene transcription; by contrast, the adjacent genes of che-lincRNAs with the opposite enrichment pattern are mainly involved in protein phosphorylation (Fig. 4G; Additional file 6: Table S5). These pathways might be essential for *in vitro*/*in planta* regeneration [7, 8]. Thus, cheRNAs could function as important components of the regulatory networks of somatic cell reprogramming.

Previous studies have showed that lncRNAs could regulate chromatin remodeling by mediating post-translational modification which is mostly related to histones [18]. For example, three lncRNAs *COLD ASSISTED INTRONIC NONCODING RNA* (*COLDAIR*), COOLAIR, and COLDWRAP regulate *FLOWERING LOCUS C* (*FLC*) transcription by mediating H3K27me3 and H3K4me3 deposition [56–59]. lncRNA *AUXIN REGULATED PROMOTER LOOP* (*APOLO*) and *MARNERAL SILENCING* (*MARS*) negatively regulate H3K27me3 deposition [60, 61], whereas NAT-lncRNA *MADS AFFECTING FLOWERING4* (*MAS*) [62] and lncRNA *LRK Antisense Intergenic RNA* (*LAIR*) [63] positively regulate H3K4me3 deposition. Intriguingly, our data showed that che-lncRNAs are positively correlated with the active histone marks H3K4me3 and H3K27ac modifications and negatively correlated with the suppressive histone mark H3K27me3 modification of their neighboring genes, whereas other histone modifications and DNA methylations are not affected by che-lncRNAs. These data implied that chromatin remodeling regulatory lncRNAs might be inclined to regulate target gene expression through mediating H3K27me3, H3K4me3, and/or H3K27ac modifications. In addition to mediate post-translational modifications, cheRNAs might also recruit transcriptional factors (TFs) to regulate gene expression, as TFs have capacity of binding snRNAs and lncRNAs [64–67].

Lastly, the extensive regeneration abilities of plants are important for their survival. Sustained stem cell activity in meristems ensures that plants undergo unlimited growth to optimize the use of resources and to heal local damage via tissue regeneration [8, 48, 49]. Thus, cheRNAs involved in somatic cell reprogramming could also play roles in organ development and stress responses. We indeed observed correlations between cheRNAs and crop traits by performing large-scale analysis of the phenotypes of mutants and transgenic plants. Most of these cheRNAs regulate tissue size, including seed size and panicle size, which are essential for grain yield. Considering their high conservation across rice varieties, these cheRNAs have great potential for use in crop trait improvement and crop breeding in the future.

## Conclusions

We systematically investigated cheRNAs in rice during callus induction, proliferation, and regeneration. These cheRNAs, which are highly conserved across plant species, shuttle between chromatin and the nucleoplasm during somatic cell regeneration. They regulate the expression of neighboring genes via specific RNA motifs, and mutant analysis implies they might be associated with plant size and seed morphology. Investigation of the functions of two che-lncRNAs supported their roles in *cis-*regulating, plant regeneration and rice traits regulation.

## Methods

### Extraction of chromatin-enriched RNAs

Three-gram samples (mature embryos, undifferentiated embryogenic callus, differential callus, and shoots) were ground with liquid nitrogen into fine powder and transferred into an ice-cold 50 ml tube with 20 ml cell lysis buffer (20 mM Tris-HCl, pH 7.4, 20 mM KCl, 2 mM EDTA, 2.5 mM MgCl_2_, 25% glycerol, 250 mM sucrose, and 5 mM DTT, cocktail plant protease inhibitor, 5 U/ml RNase inhibitor). After homogenization by vortexing, the extracts were kept on ice for 15 min. Then, the homogenate was filtered through two layers of Miracloth. After centrifugation at 4 °C and 2500*g* for 10 min, the supernatant was removed and collected as the cytoplasmic fraction for western blot, and the pellet was resuspended and washed once with 2 ml cell lysis buffer. The pellet was then resuspended in 5 ml resuspension buffer (20 mM Tris-HCl, pH 7.4, 25% glycerol, 2.5 mM MgCl_2_, 0.2% Triton X-100, and5 mM DTT, 1 U/ml RNase inhibitor) and centrifuged at 4 °C, 2500*g* for 10 min. The pellet was washed three times using resuspension buffer. The supernatant was completely removed, and the nuclei were resuspended with 500 μl gradient buffer 1 (10 mM Tris-HCl, pH 8.0, 250 mM sucrose, 10 mM MgCl_2_, 1% Triton X-100, and 5 mM β-mercaptoethanol, cocktail plant protease inhibitor, 10 U/ml RNase inhibitor). A 2-ml tube with round bottom was prepared, and 500 μl gradient buffer 2 (10 mM Tris-HCl, pH 8.0, 1.7 M sucrose, 2 mM MgCl_2_, 0.15% Triton X-100, and 5 mM β-mercaptoethanol, cocktail plant protease inhibitor, 10 U/ml RNase inhibitor) was added. Gradient buffer 1 containing samples was transferred carefully on the top of gradient buffer 2 and centrifuged at 4 °C for 10 min at 12000 rpm. The supernatant was thoroughly discarded and resuspended with 500 μl glycerol buffer (20 mM Tris-HCl, pH 7.9, 75 mM NaCl, 0.5 mM EDTA, 50% glycerol, 0.85 mM DTT, 0.125 mM PMSF, 10 mM β-mercaptoethanol, and 125 U/ml RNase inhibitor). The suspension was transferred into 500 μl urea buffer (10 mM HEPES, pH 7.6, 7.5 mM MgCl_2_, 0.2 mM EDTA, 0.3 M NaCl, 1 M urea, 1% NP-40, 1 mM DTT, 0.5 mM PMSF, cocktail plant protease inhibitor, 10 mM β-mercaptoethanol, and 125 U/ml RNase inhibitor), vortexed, and kept in ice for 5 min. It was then centrifuged at 4 °C, 13,000 rpm for 2 min, and the supernatant was collected as the nucleoplasmic fraction. The pellet was washed again with glycerol buffer and urea buffer as mentioned above. The pellet was retained as the chromatin fraction. Several nucleoplasmic and chromatin fractions were collected for western blot analysis.

For RNA extraction, the pellet was resuspended in 1 ml TRIzol. The nucleoplasmic fraction was mixed with 2.632 volumes of RNA precipitation solution (ethanol containing 0.15 M sodium acetate, pH 5.5), vortexed thoroughly, and kept at − 20 °C overnight. The pellet was vortexed and centrifuged at 4 °C, 18,000*g* for 15 min. The supernatant was discarded and the pellet air-dried. Then, 1 ml TRIzol was added to lyse the pellet. Two hundred microliters of chloroform was then added, vortexed for 10 s, and kept at room temperature for 5 min. The mixture was centrifuged at 4 °C and 12,000*g* for 15 min. The supernatant was transferred to a new tube, and 1.5 volume of GXP2 buffer (HiPure HP Plant RNA Mini Kit, Magen, China) was added. The solution was vortexed and transferred into a Spin Column (Plant/Fungi Total RNA Purification Kit, NORGEN, Canada). The extraction procedures were performed according to the manufacturer’s instructions (Plant/Fungi Total RNA Purification Kit, NORGEN, Canada). The RNA samples were quantified using a Nanodrop 2000 and stored at − 80 °C.

To verify the purity of each fraction, the total protein, cytoplasmic, nucleoplasmic, and chromatin protein fractions were subsequently analyzed using western blot. For immunoblot analysis, antibodies against GAPDH (BPI, AbP80006-A-SE) and Histone H3 (Abcam, Ab1791) were used for cytoplasmic and chromatin fraction-specific markers, respectively.

### Library construction and sequencing

The extracted RNA was prepared for RNA sequencing and deep sequencing of intermediate-size RNAs (50 to 300 nt) with two biological replicates. For RNA-seq, the total RNA quantity and purity were analyzed using a Bioanalyzer 2100 and RNA 6000 Nano LabChip Kit (Agilent, CA, USA) with RIN number > 7.0. The preparation of whole-transcriptome libraries and deep sequencing were performed by the Annoroad Gene Technology Corporation. Libraries were controlled for quality and quantitated using the BioAnalyzer 2100 system and qPCR (Kapa Biosystems, Woburn, MA). The resulting libraries were sequenced initially on a HiSeq 2000 instrument that generated paired end reads of 150 nt. For intermediate-size RNA-seq, the library size selection was performed by gel electrophoresis with a range of 50–300 bp. Approximately 1 μg of total RNA was used to prepare the library according to the protocol of the TruSeq Small RNA Sample Prep Kits (Illumina, San Diego, USA). The libraries were subsequently sequenced on the Illumina HiSeq2500 platform at LC-BIO (Hangzhou, China) following the manufacturer’s instructions, and the full-length pair-end reads were obtained. The datasets generated during the current study are available in the SRA database of NCBI (SRP338667) [68].

### Sequencing data processing and novel ncRNA identification

For transcriptome sequencing data, the read quality was inspected using FastQC v0.11.9 and then aligned to the *Oryza sativa* genome assembly (MSU RGAP Release 7 [69]) using TopHat v2.1.1 [70]. The transcript from each dataset was de novo assembled independently using Cufflinks v2.2.1 [71]. The CPE and SNE transcripts from all samples were pooled and merged to generate a single final GTF file using the Cuffmerge program, and the abundance of all transcripts was estimated by Cuffdiff based on the final GTF file.

For intermediate-sized RNA sequencing data, the adapters in raw reads were removed using Cutadapt v3.0 [72], and the untrimmed paired reads were merged using PEAR v0.9.6 [73], combining the trimmed first-end reads into single read FastQ files. Reads were then aligned against intermediate-size ncRNAs for perfect matches using the STAR v2.7.5 [74] program with the following priority: rRNA (RAP-DB [75]), tRNA (RAP-DB), snRNA (Rfam database v14.5 [76]), and annotated snoRNA (Rfam database v14.5 and published articles [27]). Reads that could not be mapped to either class above were converted into FastA format using the fastx_collapser program from FASTX-Toolkit v0.0.14 (http://hannonlab.cshl.edu/fastx_toolkit/index.html) and then aligned to genome assembly using Bowtie v2.4.1 [77]. The novel snoRNAs were identified using snoSeekerNGS-1.0 [78] against the alignment files, and the prediction results were gathered and filtered with the conserved box motif, resulting in the novel snoRNA candidates. All the mapped reads above were then aligned to the annotated intermediate-size ncRNAs and novel snoRNA candidates and filtered by the length coverage using the manual Perl script. The effective reads were aligned to the genome and counted using featureCounts v2.0.1 [79]. Only ncRNAs with more than 3 supported reads in at least 2 samples or more than 10 supported reads in at least 1 sample were kept. The expression quantity of the intermediate-size ncRNAs was normalized by RPM (reads per million).

Raw count matrixes were counted by featureCounts, and differential expression analysis was performed by DEseq2 v1.32.0 [80] in R (version 4.1.0), setting an adjusted *p* value less than 0.05 as the cutoff for statistical significance. The ncRNAs with CPE versus SNE fold change > 1.2 were classified as chromatin-enriched RNAs (che-RNAs), while those with fold change < 0.8 were classified as soluble nuclear extract enriched RNAs (sne-RNAs). The che-lincRNAs and che-lncNATs were identified by estimating the coding potential using CPC2 [81] and comparing the genomic coordinate and strand with the MSU7.0 annotated mRNA transcripts using intersectBed (bedtools v2.29.2 [82]). The possible TEs derived che-lincRNAs (TE che-lincRNA) were identified by overlapping with known rice TEs. The rice TE annotations used in this study are obtained from the outputs of the RepeatMasker which were filtered to remove some non-TE elements, including low complexity, satellites, simple repeats, and ncRNAs. Identified cheRNAs transcripts were extracted and added to the MSU7.0 mRNA annotation, and the expression abundance in Input samples was estimated using Cuffdiff.

### Constructs for genetic transformation

To construct the RNAi transformation plasmid, 300 nt DNA fragments of chr8 22101135 to 22100836 for *OsCHELIN2084* and chr6 23891471 to 23898765 for *OsCHENAT1709* were ligated to modified pRTV vector [83]. And the pRHCas9 vector [83] was used to construct the knock out mutant. The sgRNA target sites by CRISPR-cas9 are chr8 22107716 to 22107735 for LOC_Os08g35070 and chr8 22111291 to 22111310 for LOC_Os08g35090 respectively.

### Chromatin RNA immunoprecipitation (ChRIP)

1.5 g callus and shoot were crosslinked in 30 ml of 1.0% formaldehyde under vacuum for 30 min in a desiccator attached to a vacuum pump. Then, quench cross-linking in 0.125M Glycine solution for an additional 5 min was done. Wash the samples with distilled water three times, and then ground the samples into fine powders. Nucleus were isolated, lysed and sonicated into 1 kb fragments, immunoprecipitated with histone H3 antibody (Abcam) or with IgG (Millipore). The chromatin-associated RNA was extracted using TRIzol (Invitrogen, USA), and DNase I treatment was conducted to remove DNA contamination. Then, the chromatin-associated RNA was reverse-transcribed into cDNA and qPCR reactions were performed for RNAs of interest using H3 and IgG pull-down fractions.

### Analysis of cheRNA neighboring gene expression and genomic features

Comparisons of neighboring gene were performed using closestBed (bedtools v2.29.2) with MSU7.0-annotated non-TE mRNA transcripts relative to different genomic features. The average FPKM expression of input samples was used, and all boxplots were plotted using the R package ggplot2 v3.3.3 in R. The Pearson correlation coefficient (PCC) was calculated between the expression levels of cheRNAs and their neighboring genes in R. The *p* values were calculated using a Wilcoxon Mann-Whitney test.

### Analysis of epigenetic activities

The H3K27ac, H3K4me3, H3K27me3 ChIP-seq [84], and DNase-seq [31] analyzed data were downloaded from Plant Chromatin State Database (PCSD) [85]. The raw sequencing data of H4K12ac and H3K9me2 ChIP-seq and Bisulfite sequencing (BS-seq) were downloaded from the NCBI database (PRJNA386513 [86], PRJNA142153 [31], GSE126436 [87], GSE42410 [50]); all raw reads adapters were removed using cutadapt. The ChIP-seq reads were aligned to the *Oryza sativa* genome assembly (MSU RGAP Release 7 [49]) using bowtie2. The mapped reads were converted to bigwig format for visualization using bamCoverage from deeptools v3.5.1 [88]. The BS-seq reads mapping and methylation extraction were conducted using Bismark v0.23.1 [89]. The DNA methylation levels were calculated by averaging the DNA methylation ratios of all cytosine sites with coverage larger than 5 in 20 bp windows. All region profiles were computed and plotted using deeptools v3.5.1 commands. All other public datasets used in the study were listed in Additional file 4: Table S3.

### Clustering and Gene Ontology analysis

The CPE versus SNE fold change of cheRNAs was defined as the chromatin enrichment score and used to perform a time-series cluster with a series of embryo, callus, differentiated callus, and shoot sample. The time-series soft clustering analysis was conducted by the fuzzy *c*-means method in the Mfuzz [90] package v2.52.0 to identify the different chromatin enrichment variation patterns. The neighboring genes of cheRNA with different chromatin enrichment patterns were extracted, and a GO enrichment analysis was performed with AgriGOv2 [91] (http://systemsbiology.cau.edu.cn/agriGOv2/). The significant GO enrichment results (*p* < 0.05) were summarized using the REVIGO [92] website (http://revigo.irb.hr/). The aggregate size indicates the significance levels of the GO terms, as determined using the Yekutieli test with false discovery rate correction. PCA (principal component analysis) was conducted with the normalized abundance FPKM of indicated RNAs using the R package factoextra v1.0.7 and plotted by ggplot2.

### Whole-genome alignment and conservation analysis

Pairwise whole-genome alignments with the *Oryza sativa* japonica genome were generated for each *Oryza* species following the UCSC pipeline. Specifically, the other *Oryza* species genomes were downloaded from the NCBI Assembly database, including *O. sativa* indica group (PRJNA353946), *O. rufipogon* (PRJEB4137), *O. nivara* (PRJNA48107), *O. barthii* (PRJNA30379), *O. glaberrima* (PRJNA13765), *O. glumaepatula* (PRJNA48429), *O. meridionalis* (PRJNA48433), *O. punctata* (PRJNA13770), *O. brachyantha* (PRJNA70533), and *L. perrieri* (PRJNA163065). All the repetitive DNA was masked from genomes using RepeatMasker v4.0.8. Each pairwise alignment was conducted using the RunLastzChain.sh script from UCSC Kent Utils setting a “Near” parameter, and the Netting and Maffing steps were performed using the UCSC pipeline program with manual scripts. All of the above computations were run in parallel in a Linux cluster. The reference-guided multiple alignments were conducted by the Roast v3 program [93]. A phylogenic model was fitted based on the multiple alignment of the 11 *Oryza* genomes using the phyloFit program [94]. The conservation scores of each base were calculated from the 11-way alignments based on the fitted model using the phastCons program.

### SnoRNA genome organization and target RNA prediction

The identified novel snoRNAs were combined with all annotated snoRNAs, and snoRNA clusters and genome organization were determined as previously described in the literature [27]. SnoRNAs with less than 500-bp gene intervals were classified into the same cluster. All genomic, family, and chromatin enrichment information of all expressed snoRNAs was gathered and plotted into a sankey diagram using the ggplot2 extension ggforce in R. The snoRNA modification targets were predicted by PLEXY [95] for CD box snoRNAs and RNAsnoop [96] v2.4.17 for HACA box snoRNAs, and the target RNA sequences (rRNAs and snRNAs) were obtained with the annotation downloaded from the RAP-DB and Rfam databases.

### Insertion mutant and crop trait–associated SNP analysis

The T-DNA insertion mutant analysis was performed as previously described [97]. The T-DNA insertion site of *OsCHELIN2084* is at chr8 22101165, and the T-DNA insertion site of *OsCHENAT1709* is at chr6 23896685. The crop trait–associated SNP GWAS (Genome Wide Association Study) data were downloaded from the Rice SNP-Seek Database [42] (https://snp-seek.irri.org/_gwas.zul ), setting the minimum -log_10_(*p* value) as 4 and the subpopulation option as “all varieties.” The crop trait–associated SNP genomic locations were compared with the che-lincRNAs’ genomic coordinates using intersectBed.

### Triple helix formation prediction and motif analysis

The potential DNA:DNA:RNA triple helix sites of che-lincRNAs were predicted using Triplexator v1.3.2 [98]. The predictions were performed with the parameters of “-l 20 -e 5” and other defaulted parameters. The triplex-forming target site DNA regions were annotated and plotted using the ChIPseeker [99] v1.28.3 package across the MSU7.0 gene annotation. The triplex formation of che-lincRNAs and their neighboring gene DNA regions was tested using the Triplex Domain Finder region test program [100] (rgt-TDF v0.13.2 from the Regulatory Genomics Toolbox).

De novo motif analysis was conducted using the MEME suite [101] v4.11.2. The motif distributions were scanned using Fimo from the MEME suite, and the relative location was calculated and normalized for each transcript length. The density distribution was plotted using ggplot2 in R.

### Total RNA extraction and qRT-PCR

Total RNA extraction and qRT-PCR were performed as described previously [97]. The results were presented as the relative expression levels normalized to the expression of *OsActin2*. For the semi-quantitative RT-PCR analysis, the amplification was performed in a 20-μl reaction volume containing diluted cDNA, 0.4 mM primers, diethylpyrocarbonate-treated water, and TB Green® Premix Ex Taq^TM^ (TAKARA). The PCR conditions were as follows: 95 °C for 30 s, followed by various cycles according to different genes of 95 °C for 10 s. The PCR products were electrophoresed in a 2.5% agarose gel, and the images were captured. Each qRT-PCR was performed for three biological replicates. The primers used for both semi-quantitative RT-PCR and qRT-PCR are listed in Additional file 9: Table S8.

### Tissue culture procedure

Mature, healthy seeds were sterilized by immersion in 70% ethanol for ~ 2 min, followed by 2.5% sodium hypochlorite solution for 30 min with shaking, and rinsed five or six times with sterile water on an ultraclean workbench. N6 was used as the main callus induction medium, and 2 mg/L 2,4-D and 30 g/L sucrose were added. The pH of the medium was adjusted to 5.8, and 3.0 g/L phytagel was added to the medium before boiling. Approximately 90 mature seeds per line, evenly distributed among two dishes, were incubated in induction medium for 15 days at 28 °C. The induced calli were transferred to subculture medium and incubated at 28 °C for 15 days. After 1 month culture, the calli were transferred to differential medium (MS, 2 mg/L 6BA, 2 mg/L KT, 0.2 mg/L IAA, 0.2 mg/L NAA, and 30 g/L sucrose; the pH of the medium was adjusted to 5.8, and 3.0 g/L phytagel was added to the medium before boiling) and incubated for 30 days at 28 °C.

### Phenotype observations

Images of calli during the induction stage were taken by a LEICA M205FA (Germany). The images were taken after the calli were transferred to subculture medium for 0, 5, 10, and 15 days, and the sizes were calculated using ImageJ by measuring the mean size.

### Scanning electron microscopy

To prepare histological sections, calli that had been cultured for 25 days were fixed in FAA fixative solution (50% alcohol: acetic acid: formaldehyde = 89:6:5) for 30 min under vacuo, and post-fixed in the same buffer overnight. After being dehydrated through an ethanol series and dried using a carbon dioxide critical-point dryer, the calli were cleaned with ethanol and dried at 45 °C. The dry calli were gold plated and photographed under a Hitachi S-3400 N scanning electron microscope (Japan).

## Supplementary Information

**Additional file 1: Supplementary Figures 1-6.**

**Additional file 2: Table S1.** Sequencing information.

**Additional file 3: Table S2.** Information of cheRNAs.

**Additional file 4: Table S3.** Public datasets used in this study.

**Additional file 5: Table S4.** Neighboring genes which significantly correlated with cheRNAs.

**Additional file 6: Table S5.** The predicted DNA binding sites of che-lincRNAs.

**Additional file 7: Table S6.** GO terms of the neighboring genes of the che-lincRNAs from different clusters.

**Additional file 8: Table S7.** phenoype analysis of the cheRNA T-DNA insertion mutants.

**Additional file 9: Table S8.** Primers used for qRT-PCR and plasmid construct.

**Additional file 10.** Uncropped images.

**Additional file 11.** Review history.

## Data Availability

The datasets generated during the current study are available in the SRA database of NCBI (SRP338667) [68]. The published data used in this study were downloaded from the NCBI database (PRJNA386513 [86], PRJNA142153 [31], GSE126436 [87], GSE42410 [50].
