# Supplementary material for: Genome-wide analysis and functional annotation of chromatin-enriched noncoding RNAs in rice during somatic cell regeneration
Source: Genome Biol. 2022 Jan 19;23:28. doi: 10.1186/s13059-022-02608-y (PMC8772118; doi:10.1186/s13059-022-02608-y)
Supplement: Supplementary file 1 — Additional file 1: Supplementary Figures 1-6. [file 13059_2022_2608_MOESM1_ESM.docx]

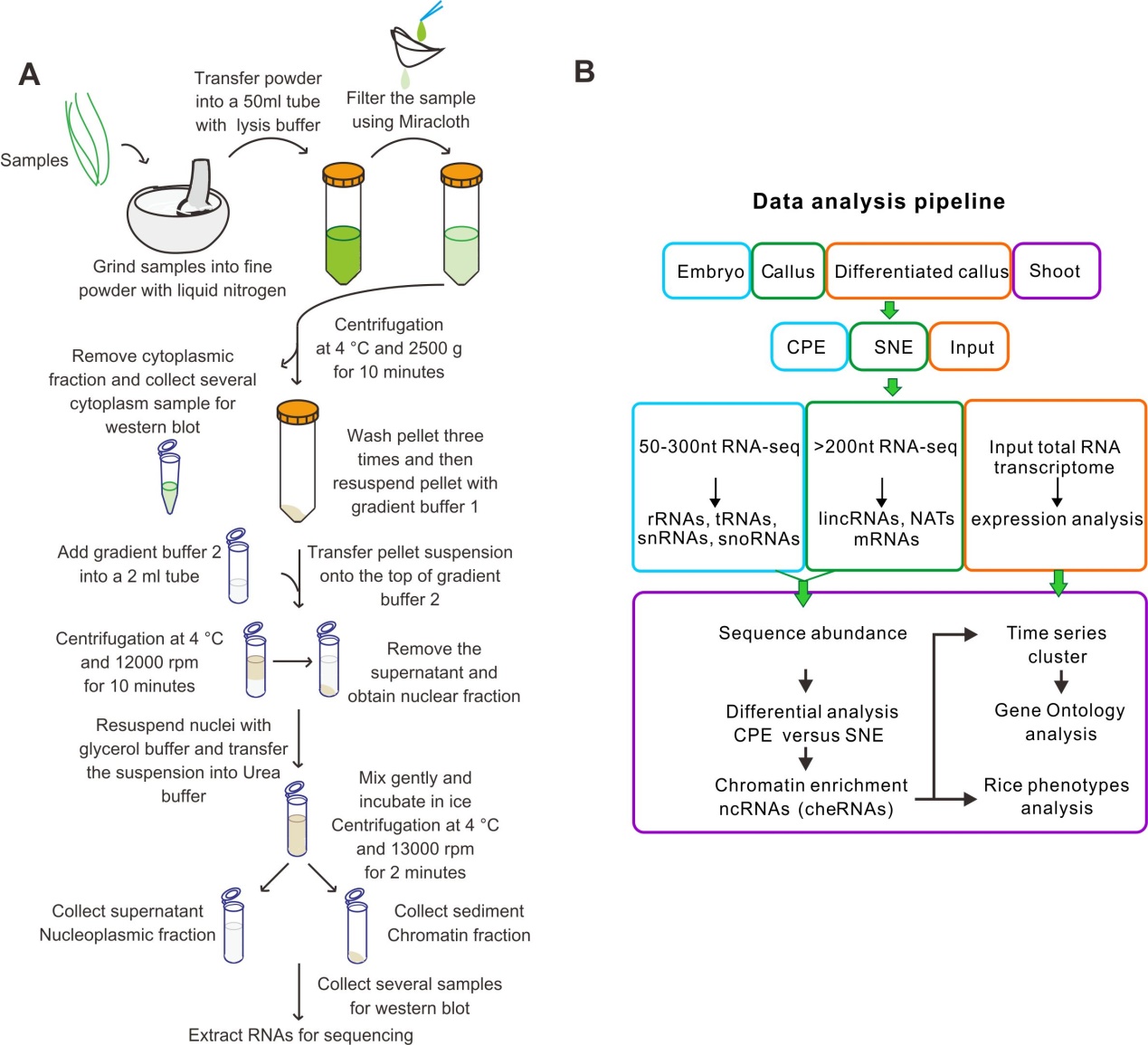
**Supplementary Figure 1. Schematic representations of the procedures used for nuclear fractionation experiments, sequencing, and data analysis.** (**A**) Schematic representation of the nuclear fractionation procedure. (**B**) Schematic representation of the sequencing and data analysis procedures.


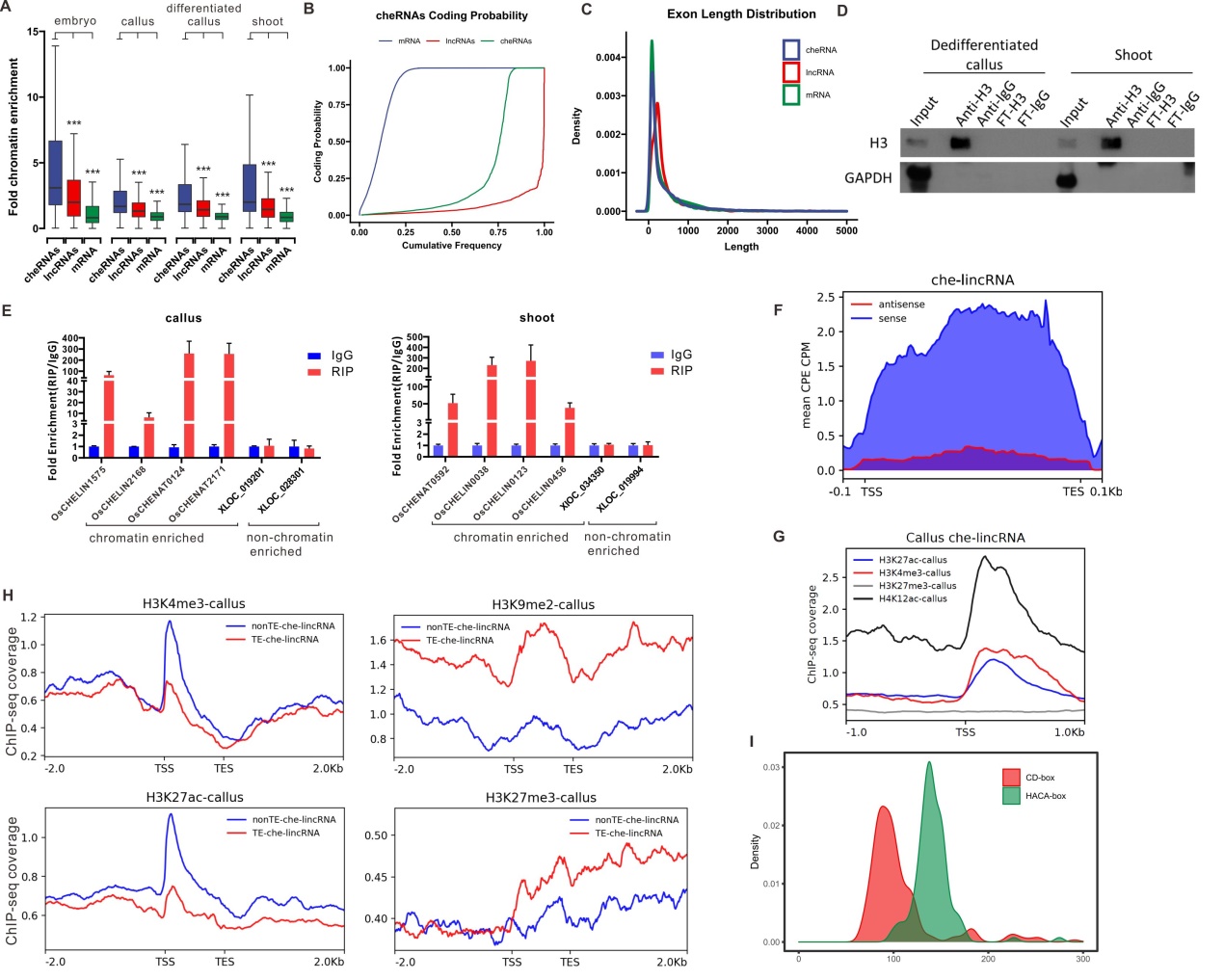


**Supplementary Figure 2. Properties of rice cheRNAs.** (**A**) Analysis of fold enrichment of the indicated RNA classes on chromatin; *p* values were calculated by Wilcoxon Mann-Whitney test. Values are the means ± SD. *p* values were calculated by *t*-test, *p* < 0.05 (*), *p* < 0.01 (**), *p* < 0.001 (***) (**B**) The cumulative coding potentials of mRNAs (annotated using MSU7.0), lncRNAs (annotated using NONCODEv6), and cheRNAs predicted using the CPC2 program. (**C**) Density plot of exon length distributions for cheRNAs, lncRNAs (NONCODEv6 annotated), and mRNAs (annotated using MSU7.0). (**D**) Immunoblot analysis of histone H3 and GAPDH in input, chromatin, and IgG fraction of ChRIP assay. FT means flow through. (**E**) qRT-PCR analysis of the fold enrichment of cheRNAs in callus and shoot. The left panel exhibited the cheRNAs which are chromatin enriched in callus (chromatin enriched); the right panel exhibited the cheRNAs which are chromatin enriched in shoot (chromatin enriched). Four lncRNAs which were identified in nucleoplasm were used as negative control (non-chromatin enriched). (**F**) Average coverage of stranded RNA-seq reads from combined CPE replicates that map to cheRNAs gene region. Coverage in the same sense as the cheRNA annotation is depicted in blue and antisense reads in grey. (**G**) Mean ChIP-seq coverage in callus of H4K27ac, H3K4me3, H3K27me3, H4K12ac profiles centered at the putative TSSs of che-lincRNAs. (**H**) Mean ChIP-seq coverage in callus of H3K4me3, H3K9me2, H4K27ac, and H3K27me3 profiles scaled the gene region of the TE che-lincRNAs and non-TE che-lincRNAs. (**I**) Density plot of transcript length distributions for intermediate-sized che-snoRNAs, grouped by family..


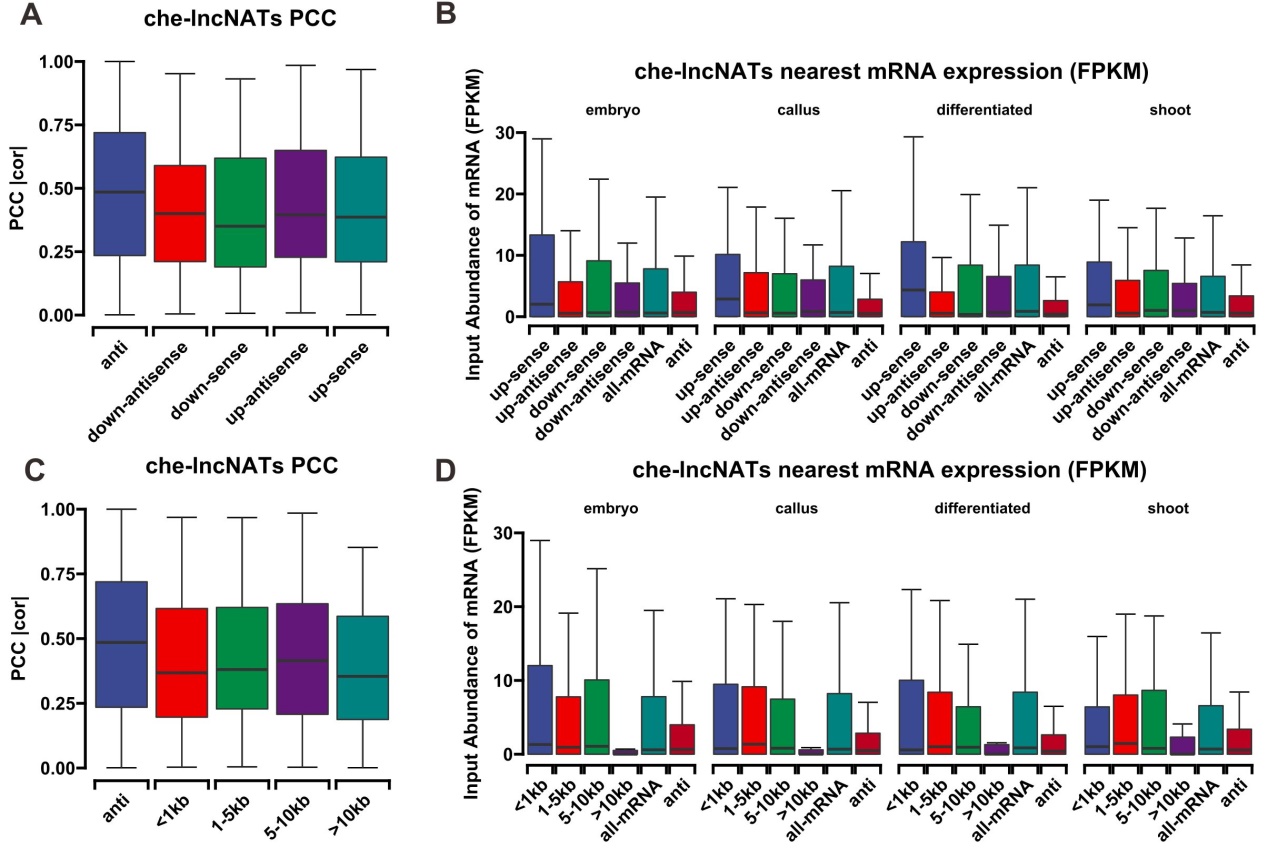


**Supplementary Figure 3. Analysis of the regulatory mechanism of che-lncNATs.** (**A**) Comparison of the Pearson correlation coefficient (PCC) absolute values of the expression of che-lncNATs and their neighboring genes in input, grouped based on strand and orientation to che-lncNATs. *p* values were calculated by Wilcoxon Mann-Whitney test. (**B**) Comparison of input expression (FPKM) of the nearest neighboring genes reflecting strand and orientation to che-lncNATs in four tissues. (**C**) Similar to (A) but grouped by distance to che-lncNATs. (**D**) Similar to (B) but grouped by distance to che-lncNATs.


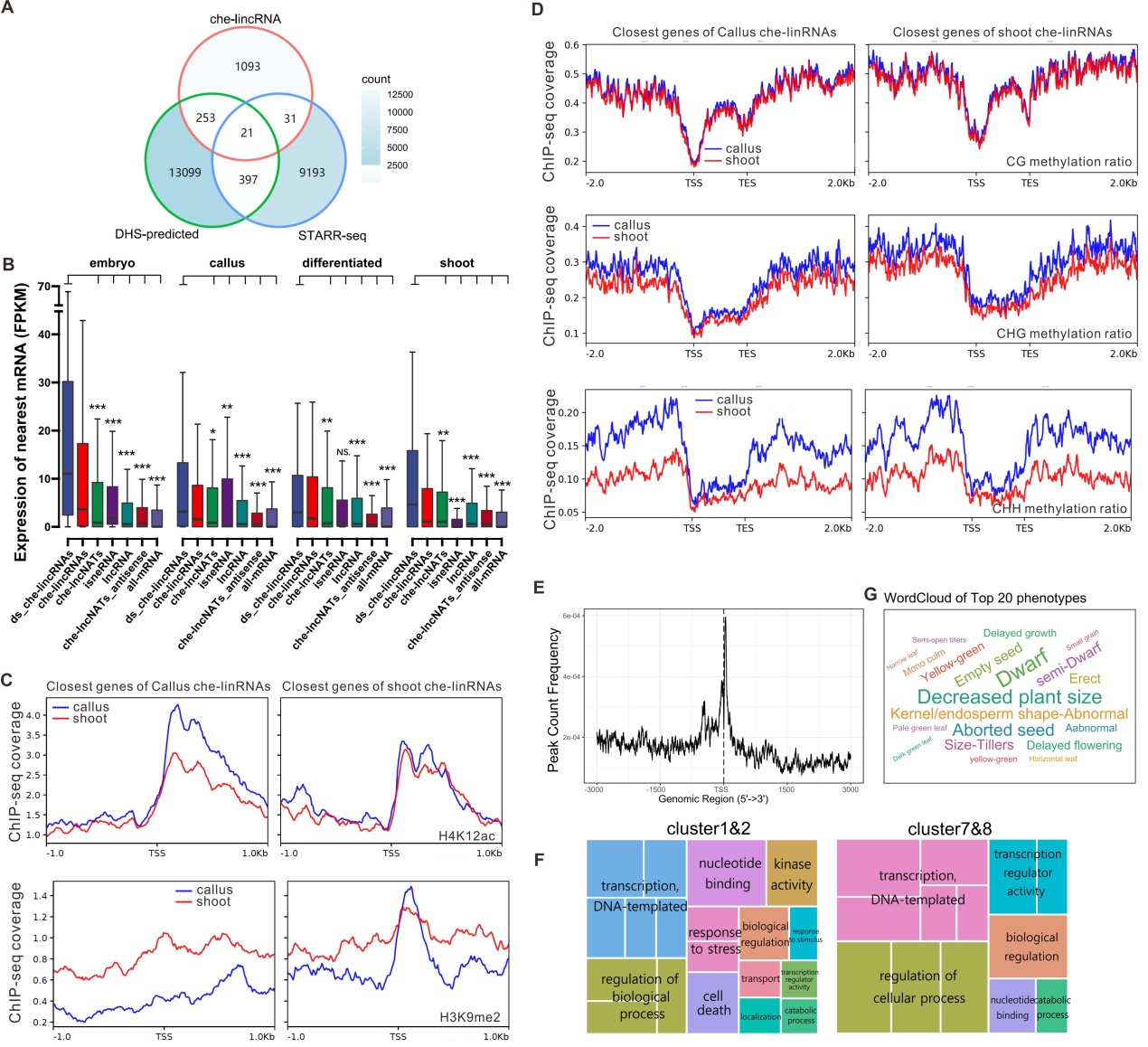


**Supplementary Figure 4. Functional analysis of cheRNAs.** (**A**) Venn diagram of the overlap of che-lincRNAs genomic coordinates with enhancers predicted by DHS and STARR-seq. (**B**) Comparison of input expression (FPKM) of the nearest genes to the indicated genomic features: che-lincRNAs downstream and in the same sense as their neighbors (ds-sense che-lincRNAs), all che-lincRNAs, che-lncNATs, intergenic SNE-enriched lncRNAs, lncRNAs (annotated using NONCODEv6), antisense with che-lncNATs, and all mRNAs; *p* values were calculated by Wilcoxon Mann-Whitney test, *p* < 0.05 (*), *p* < 0.01 (**), *p* < 0.001 (***). (**C**) Mean ChIP-seq coverage in callus and shoot of H4K12ac and H3K9me2 profiles centered at the TSS of the closest genes of the closest genes of callus-specific or shoot-specific enriched che-lincRNAs respectively (mean CPE FPKM ≥ 5). (**D**) DNA methylation level profiles of the CG, CHG and CHH context in callus and shoot scaled the region around the closest genes of the closest genes of callus-specific or shoot-specific enriched che-lincRNAs respectively (mean CPE FPKM ≥ 5). (**E**) Average profile of the genomic distribution of the predicted triplex forming target sites. TSS, transcript start site. (**F**) GO analysis of neighboring genes of che-lincRNAs from the indicated clusters. The significant GO enrichment results (*p* < 0.05) were summarized using REVIGO. The aggregate size indicates the significance levels of the GO term, as determined using the Yekutieli test with false discovery rate correction. (**G**) Word cloud of the top 20 most frequent che-lincRNA T-DNA mutant phenotypes; the word size represents the frequency.


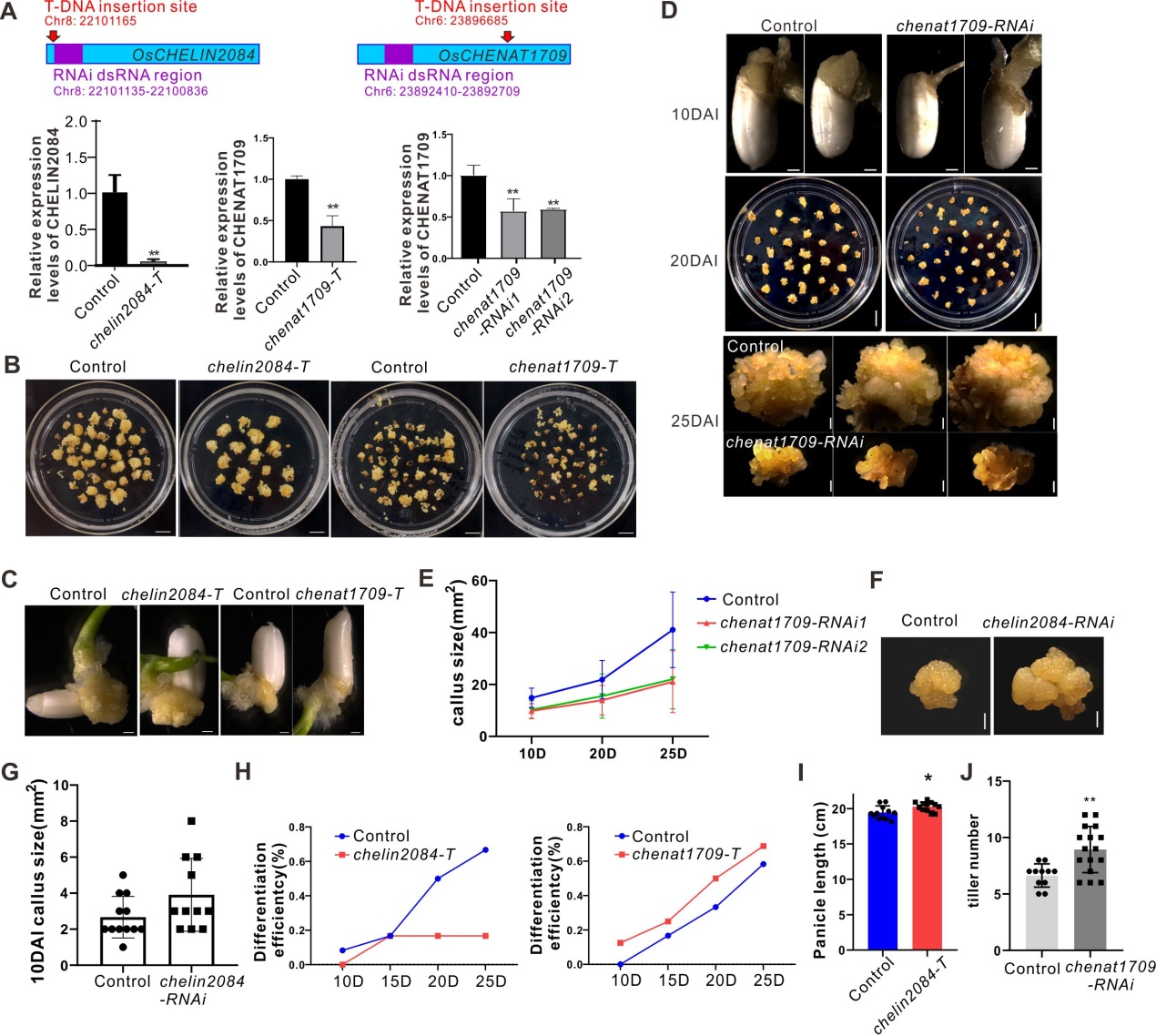


**Supplementary Figure 5. Functional analysis of two cheRNAs.** (**A**) The information of the T-DNA insertion mutant and the RNAi transgenic plants of *OsCHELIN2084* and *OsCHENAT1709*; and the relative expression level of *OsCHELIN2084* and *OsCHENAT1709* in *chelin2084*-*T*, *chenat1709-T* and *chenat1709-RNAi* plants. Values are the means ± SD (*n* = 3 replicates)；*p* values were calculated by Wilcoxon Mann-Whitney test, *p* < 0.05 (*), *p* < 0.01 (**). (**B**) Callus at 30 days after induction in DJ-WT, *chelin2084*-*T*, ZH11-WT, and *chenat1709-T* from left to right. Scale bars, 1 cm. (**C**) Differentiated callus at 10 days after differentiation from DJ-WT, *chelin2084*-*T*, ZH11-WT and *chenat1709-T* from left to right. Scale bars, 1 mm. (**D**) Callus at 10, 20 and 25 days after induction (DAI) in empty-vector control and *chenat1709-RNAi* transgenic plants. Scale bars, 1 cm for 20 DAI callus; 1mm for 10 DAI and 25 DAI callus. (**E**) Callus size of empty-vector control plants and *chenat1709-RNAi* transgenic plants from two lines during callus induction (right panel). Values are the means ± SD (*n* = 31, 41, 43 callus for vector control, RNAi-1, and RNAi-2 respectively). (**F**) Callus transferred with empty-vector or *chelin2084-RNAi* vector at 15 DAI. Scale bars, 1 mm; (**G**) Callus size of callus transferred with empty-vector or *chelin2084-RNAi* vector at 10 DAI. Values are the means ± SD (*n* = 12 and 11 callus for vector control and RNAi respectively). (**H**) Analysis of differentiation efficiency. (**I**) Statistical analysis of panicle length. Values are the means ± SD (*n* = 12 plants). *p* values were calculated by Wilcoxon Mann-Whitney test, *p* < 0.05 (*), *p* < 0.01 (**). (**J**) Tiller numbers of empty-vector control and *chenat1709-RNAi* transgenic plants. Values are the means ± SD (*n* = 15 and 18 plants for vector control and RNAi respectively); *p* values were calculated by Wilcoxon Mann-Whitney test, *p* < 0.05 (*), *p* < 0.01 (**).


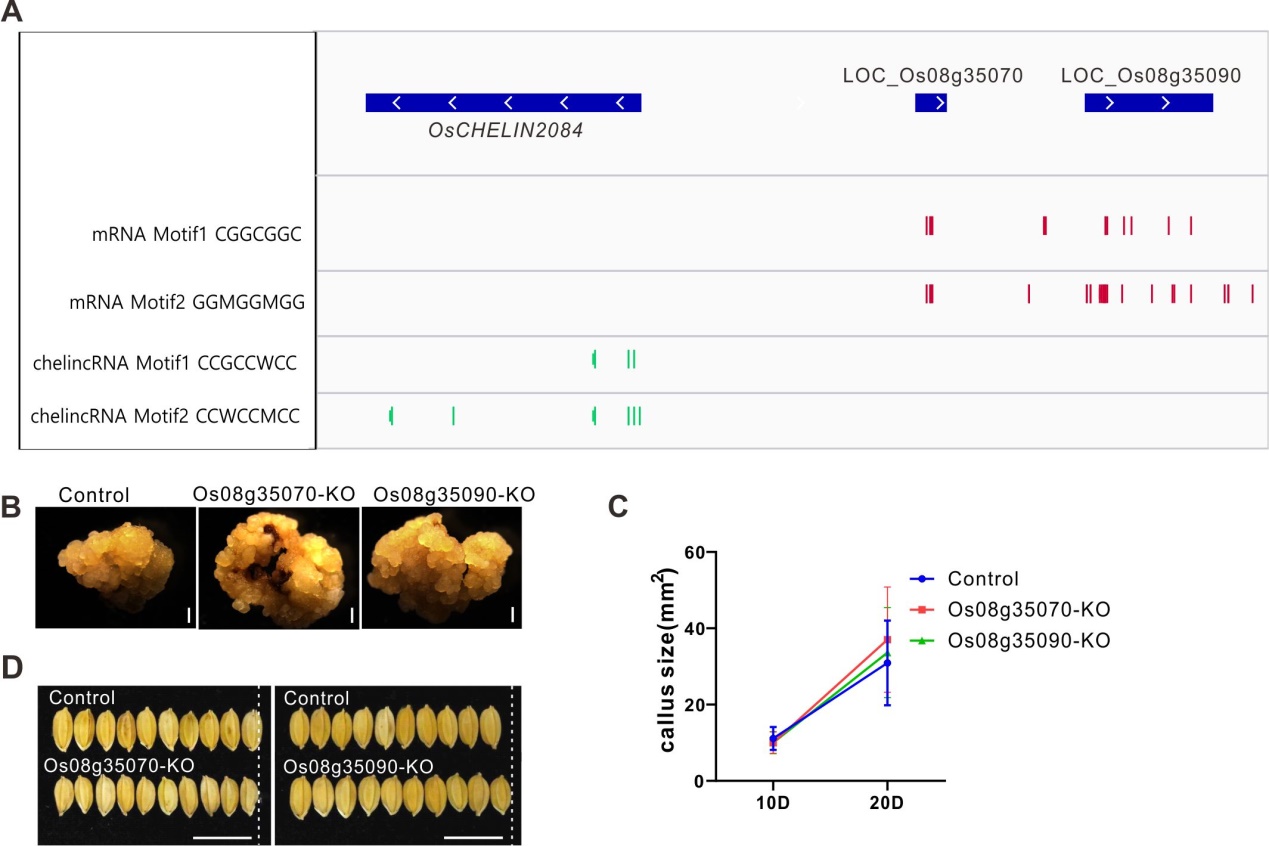


**Supplementary Figure 6. Phenotype analysis of LOC_Os08g35070, and LOC_Os08g35090.** (**A**) Gene structures and motif locations in XLOC_060928, LOC_Os08g35070, and LOC_Os08g35090. (**B**) Callus at 25 days after induction (DAI) in empty-vector control, LOC_Os08g35070 knockout (KO) and LOC_Os08g35090 knockout (KO) transgenic plants. Scale bars, 1 mm. (**C**) Callus size of empty-vector control plants, LOC_Os08g35070 knockout and LOC_Os08g35090 knockout transgenic plants during callus induction. Values are the means ± SD (*n* = 9, 82, and 72 callus for vector control, Os08g35070-KO, and Os08g35090-KO respectively). (**D**) Seeds of empty-vector control, LOC_Os08g35070 knockout and LOC_Os08g35090 knockout transgenic plants. Scale bars, 1 cm.
