# Supplementary material for: Genome-wide analysis and functional annotation of chromatin-enriched noncoding RNAs in rice during somatic cell regeneration
Source: Genome Biol. 2022 Jan 19;23:28. doi: 10.1186/s13059-022-02608-y (PMC8772118; doi:10.1186/s13059-022-02608-y)
Supplement: Supplementary file 11 — Additional file 11. Review history. [file 13059_2022_2608_MOESM11_ESM.docx]

Review History

**First round of review**

**Reviewer 1**

**Are you able to assess all statistics in the manuscript, including the appropriateness of statistical tests used?**

Yes, and I have assessed the statistics in my report.

**Comments to author:**

Emerging evidences have revealed the involvement of ncRNAs in chromatin remodeling, but there still be pretty limited information about these ncRNAs, such as their varieties and characteristics. Recently, several approaches developed to isolate chromatin-associated ncRNAs in animals have been shown with high efficiency; however, whether these strategies are available for plants is lack of demonstration. This MS is a pioneer identification of chromatin-interacted non-coding RNAs in plants, even though there are still many shortcomings on the function annotation of these ncRNAs.

The key point of the paper is the RNAs authors isolated are chromatin-bound. Although the authors provided WB to show there was no contamination of CPE and SNE, I think some experiments such as histone RIP could directly validate lncRNAs in chromatin enrichment.

The major flaw is that there is no relationship analysis between ncRNAs and epigenetic activities. Because RNAs isolated by the authors are from chromatins, these RNAs may be directly involved in DNA methylation and histone modification. As numerous data on rice histone and DNA status have been released, it is readily to compare them with the enrichment of cheRNAs. One of interesting findings is that cheRNAs positively regulate neighbor gene expressions, thus it would be more striking if the authors can establish the relationship between positive histone marks and cheRNAs.

A good example of cheRNAs function in rice is that XLOC_060928 regulates cell dedifferentiation and grain size through promote two neighboring genes. This seems reasonable, since the expression patterns of these two genes are positively correlated with XLOC_060928 accumulations. Unfortunately, the function of these two genes still keeps unknown, thus it should firstly present the role of these two target genes of XLOC_060928 in rice somatic embryogenesis and development. Also, there is lack of evidence showing the expression of indicated che-lncRNAs in their insertional mutants.

Other comments:

1. p5-6, " We detected 13,065 (80.17%) mRNAs and 3,232 (19.83%) ncRNAs as highly enriched on chromatin (Additional file 2: Supplementary Table 2). The chromatin enrichment ratio of those mRNAs was significantly lower than that of annotated lncRNAs and those chromatin-enriched ncRNAs (cheRNAs) (Fig. S2A)". It is difficult to know which is more abundant. Please clarify.

2. p6 line 57. I think only shuttling of cheRNAs between CPE and SNE cannot suggest cheRNAs as functional regulators of somatic cell reprogrammming.

3. p7 line 42 how can the authors get the conclusion that chelincRNAs are not be the byproducts of promoters or read-through transcription from upstream genes?

4. Overall, the authors highlight that diverse che-ncRNAs are developmental stage specific, such as p10 line 30, however, since a big part of che-ncRNAs sequencing data are obtained from undifferentiated and partially regenerated callus, I am not sure whether this belongs to plant development stage.

5. p15 line 55-60, it is pretty confused about the wild-type plants and the shoot primordia emerged dates for mutant1 and mutant 2, respectively.

6. figure 6, there is no statistics analysis of the wt and mutant tillers.

7. p18 line31-40, "We found... involved in signal transduction" How did the authors get this conclusion. I cannot find any data support this.

8. p19 line 18-20 "They regulate the expression of neighboring genes via specific RNA motifs, and are associated with plant size and seed morphology" Again, only one example to conclude cheRNAs critical in plant size and morphology seems to be overstated.

**Reviewer 2**

**Are you able to assess all statistics in the manuscript, including the appropriateness of statistical tests used?**

No, I do not feel adequately qualified to assess the statistics.

Comments to author:

In this work, Zhang and co-workers present the first identification, classification and partial characterization of cbromatin-associated lncRNAs, the so-called cheRNAs, in plants. To this end, they have performed RNA-seq of chromatin vs nucleoplasm fractions of previously isolated nuclei, obtained from different tissues, notable including in vitro grown calli. Based on a primary identification, the authors proceeded to a classification of novel chromatin-associated lncRNAs according to their anatomy regartding neighboring genes and including a set of correlations of transcriptional activity and potential action in cis or in trans. Finally, they characterized the physiological role of a subset of selected cheRNAs.

As an interesting reader in plant lncRNAs, I find this work important and valuable for the broad audience of GB. It is very well written and it's easy to follow. Congratulations! However, I still have a few major and minor concerns that I think that should be addressed prior to publication:

Major concerns:

1. Page 5, line 42: the authors state that all the analyses are based on 2 biological raplicates. Although the main aim of the experiment was to identify transcripts, the truth is that authors conclude about enrichment of chromatin vs nucleoplasm, etc. I want to make it clear that I'm not an expert in stats behind RNA-seq, and I strongly recommend the Editor to assess this point together with experts from Genome Biology, and/or other reviewers. A third replicate might be requested.

2. When the authors characterized the physiological role of selected lncRNAs, they used T-DNA insertional mutants. This is risky, considering the proximity of the T-DNA and neighboring genes, which may alter their expression regardless the role of the cheRNAs. Ideally, RNAi approaches (considering that CRISPR-mediated point mutations would be useless for ncRNA loci) should be included to distinguish T-DNA insertions from the lncRNA activity. Otherwise, please at least discuss better potential dual or alternative effects of these mutants.

3. When classifying the identified cheRNAs, I think the authors should include the profile of epigenetic marks (including histone modifications, DNA methylation and chromatin accessibility, eg. ATAC-seq). Notably, considering that it has been suggested that in plants TEs are the main source of active lncRNAs, how many of these elements resembles or belong to TEs and what is their epigenetic profile?

Minor comments:

I'm a bit surprised that the authors avoided in their discussion all that is known about lncRNAs and protein partners in Arabidopsis (and even in rice). From our own group, we have shown the interaction of a lncRNA, APOLO, with PRC1-like components, LHP1, regulating genes in cis (Ariel et al 2014) and in trans forming DNA-RNA duplexes, also discussed here (Ariel et al 2020). I don't say this because I expect the authors to cite specifically our papers!! But together with this, there are several lncRNAs in plants known to interact with chromatin remodelers, driving histone (de)acetylation or (de)medthylation, what could be interesting to discuss in the frame of what I suggested above in point 3. I recommend to read the last part of one of our recent reviews (Fonouni-Farde et al 2021, in "non-coding RNA") to identify these key papers. I consider that a deeper discussing would help to merge the findings with the literature.

Page 10, line 42: what do the authors think cheRNAs are doing in the nucleoplasm? Are they released from chromatin just because they are too abundant under certain circumstances or this may be a hint of dual roles in cis vs in trans? Please, discuss.

Among the different sub-classes of cheRNAs proposed by the authors, there are che-snRNAs. Strikingly, an old paper came to my mind about snRNAs regulating the capacity of TFs to bind chromatin (Higuchi et al 2007, BBA). Interestingly, the same TF (NFYA) binding snRNAs can also bind the PANDA lncRNA (Hung et al., 2011, Nat Gen). I think the authors should comment on the potential role of cheRNAs in the modulation of TFs and other chromatin-related proteins.

Additional details:

I strongly suggest the authors to give a name to each XLOC transcript they have characterized. They may be called by the same name-1, -2, -3, etc. But a name helps the community to identify who is who in the noncoding genome world!

Page 7, line 32: NONCODE is misspelled.

Line 42: "28.1% of lincRNAs are located within 500 bp of coding genes…" I think "of" should be replaced by "away from"

Line 62: what do the author mean by "traditional lncRNAs"? "Previously characterized" sounds more accurate.

**Authors Response**

**Point-by-point responses to the reviewers’ comments:**

Reviewer #1:

Emerging evidences have revealed the involvement of ncRNAs in chromatin remodeling, but there still be pretty limited information about these ncRNAs, such as their varieties and characteristics. Recently, several approaches developed to isolate chromatin-associated ncRNAs in animals have been shown with high efficiency; however, whether these strategies are available for plants is lack of demonstration. This MS is a pioneer identification of chromatin-interacted non-coding RNAs in plants, even though there are still many shortcomings on the function annotation of these ncRNAs.

*Reply: We thank you for the comments and the suggestions.*

Comment 1. The key point of the paper is the RNAs authors isolated are chromatin-bound. Although the authors provided WB to show there was no contamination of CPE and SNE, I think some experiments such as histone RIP could directly validate lncRNAs in chromatin enrichment.

*Reply: We agree. Following your suggestion, we have performed additional H3 ChRIP to validate the chromatin enrichment of 8 cheRNAs in callus (OsCHELIN1575, OsCHELIN2168, OsCHENAT0124 and OsCHENAT2171) or in shoot (OsCHENAT0592, OsCHELIN0038, OsCHELIN0123 and OsCHELIN0456). The results showed that the examined cheRNAs which are chromatin enrichment in callus or shoot were associated to H3, whereas the lncRNAs identified in nucleoplasm were not associated to H3 (see below Figure 1), to further confirm their chromatin-bound. We have added these new data to the revised manuscript (pages 6-7, lines 177-184, marked in blue) and the revised Additional file1: figure 2D, E, thank you.*

Comment 2. The major flaw is that there is no relationship analysis between ncRNAs and epigenetic activities. Because RNAs isolated by the authors are from chromatins, these RNAs may be directly involved in DNA methylation and histone modification. As numerous data on rice histone and DNA status have been released, it is readily to compare them with the enrichment of cheRNAs. One of interesting findings is that cheRNAs positively regulate neighbor gene expressions, thus it would be more striking if the authors can establish the relationship between positive histone marks and cheRNAs.

*Reply: According to the comment, we have performed additional analysis to obtain the relationship between che-lincRNAs and epigenetic activities. We have collected most of the published data on DNA methylation, histone modification and DNase-sequencing. The data on rice shoot and callus are available, whereas no data on embryo and differentiated callus was reported. Thus, we examined the DNA status and histone marks on the neighboring genes of che-linc RNAs from those data on rice shoot and callus. We found that che-lincRNAs are positively correlated with the active histone marks H3K4me3 and H3K27ac modifications and chromatin accessibility, and negatively correlated with the suppressive histone mark H3K27me3 modification of their neighboring genes, whereas other histone modifications and DNA methylations are not affected by che-lincRNAs (see below Figure 2). Interestingly, most of the reported chromatin remodeling regulatory lncRNAs regulate gene expression through mediating H3K4me3 and/or H3K27me3 modifications in plants. These data imply that chromatin remodeling regulatory lncRNAs including che-lincRNAs are inclined to regulate target gene expression through mediating H3K27me3, H3K4me and/or H3K27ac modifications. The results have been added to the revised manuscript (page 12, lines 345-353, marked in blue) and figure 4F; Additional file 1: Figure S4C, D .We appreciate the suggestion.*

Comment 3. A good example of cheRNAs function in rice is that XLOC_060928 regulates cell dedifferentiation and grain size through promote two neighboring genes. This seems reasonable, since the expression patterns of these two genes are positively correlated with XLOC_060928 accumulations. Unfortunately, the function of these two genes still keeps unknown, thus it should firstly present the role of these two target genes of XLOC_060928 in rice somatic embryogenesis and development. Also, there is lack of evidence showing the expression of indicated che-lncRNAs in their insertional mutants.

*Reply: Following your suggestion, we have performed additional experiments using the knockout transgenic plants of the two target genes of OsCHELIN2084 (LOC_Os08g35070 and LOC_Os08g35090) to observe the somatic embryogenesis process and the seed phenotypes of these transgenic plants. LOC_Os08g35070 is an Ubiquitin-protein ligase and LOC_Os08g35090 is a Subtilisin-like serine protease. As shown in the below Figure 3, knockout of LOC_Os08g35090 promoted callus proliferation and it had wider seeds than WT, which was similar with that of the loss of function mutant of OsCHELIN2084, and loss of function of LOC_Os08g35070 promoted callus proliferation, however, did not significantly affect seed sizes. Thus OsCHELIN2084 might affect somatic embryogenesis and development mainly through regulating both the two genes with different mechanism. The underlined mechanisms need further study. It has reported that Ubiquitin-protein ligase and Subtilisin-like serine protease regulate organ development[1-3], similarly, our results showed the functions of LOC_Os08g35090 on cell proliferation and seed development. These results have been added to the revised manuscript (page 18, lines 514-521, marked in blue) and Additional file 1: Figure6B-D. We have also provided the expression analysis data of che-lncRNAs in the insertional mutants as suggested (Additional file 1: Figure5A). We appreciate the comment to improve the study.*

Other comments: 1. p5-6, " We detected 13,065 (80.17%) mRNAs and 3,232 (19.83%) ncRNAs as highly enriched on chromatin (Additional file 2: Supplementary Table 2). The chromatin enrichment ratio of those mRNAs was significantly lower than that of annotated lncRNAs and those chromatin-enriched ncRNAs (cheRNAs) (Fig. S2A)". It is difficult to know which is more abundant. Please clarify.

*Reply: We have revised the sentence to “The detected chromatin enriched RNAs include 81.5% mRNAs and 18.5% ncRNAs. The chromatin-enriched ncRNAs have a significantly higher chromatin enrichment ratio than those of total mRNAs and the annotated lncRNAs, and these chromatin-enriched ncRNAs were named cheRNAs and used for the following studies”as suggested to make it clear(page 6, lines 153-159, marked in blue and Additional file 1: Figure. S2A; Additional file 2: Supplementary Table 2), thank you.*

2. p6 line 57. I think only shuttling of cheRNAs between CPE and SNE cannot suggest cheRNAs as functional regulators of somatic cell reprogramming.

*Reply: We appreciate the comment and have rephrased the sentence to “These results suggest that cheRNAs are under controlled during somatic cell reprogramming” (page 7, lines 191-192, marked in blue).*

3. p7 line 42 how can the authors get the conclusion that chelincRNAs are not be the byproducts of promoters or read-through transcription from upstream genes?

*Reply: We understand the reviewer’s concern. To address this issue, we have performed additional analysis by examining the transcription bias and the histone modification patterns around the putative transcription start sites (TSSs) of che-lincRNAs. The results showed that che-lincRNAs exhibited specific strand bias from their putative TSSs. The H3K4me3, H3K27ac and H4K12ac modifications showed enrichment at the TSSs of che-lincRNAs (see below Figure 4), which was similar with the reported transcribed genes. Together, these results supported that che-lincRNAs might not be the byproducts of read-through transcription from upstream genes. The data has been added to the revised manuscript (page 8, line 215-219, marked in blue) and Additional file 1: Figure S2F, G, thank you.*

4. Overall, the authors highlight that diverse che-ncRNAs are developmental stage specific, such as p10 line 30, however, since a big part of che-ncRNAs sequencing data are obtained from undifferentiated and partially regenerated callus, I am not sure whether this belongs to plant development stage.

*Reply: Thank you for the suggestion. For the regenerated callus, we have only collected the greenish part of callus and thus they were mainly consisted of regenerated cells. In addition, we have also used embryo and regenerated shoot for comparison, we think that the four samples represent four different developmental stages. According to your comment, we have revised the related sentences and used “tissue specific” instead of “developmental stage specific” to make it more precise (page11, lines 304 and 308; marked in blue). Thank you.*

5. p15 line 55-60, it is pretty confused about the wild-type plants and the shoot primordia emerged dates for mutant1 and mutant 2, respectively.

*Reply: According to the comment, we have revised the sentences to “Green spots emerged much earlier in mutant 2 than that of WT plants, whereas chelin2084-T (mutant 1) showed opposite, green spots formed later that that of WT plants” to make it clear (pages 16-17, lines 479-481, marked in blue), thank you.*

6. figure 6, there is no statistics analysis of the wt and mutant tillers.

*Reply: The statistical analysis of tiller number has been provided in the revised figure 6E, thank you.*

7. p18 line31-40, "We found... involved in signal transduction" How did the authors get this conclusion. I cannot find any data support this.

*Reply: We apologize for not clearly describing the data, this sentence described the enriched GO terms of the proximal coding genes of che-lincRNAs from cluster 5&6 or cluster 3&4. We have revised the sentence to make it clear (page 19, lines 569-570; page 20, lines 571-573; marked in blue), thank you.*

8. p19 line 18-20 "They regulate the expression of neighboring genes via specific RNA motifs, and are associated with plant size and seed morphology" Again, only one example to conclude cheRNAs critical in plant size and morphology seems to be overstated.

*Reply: We appreciate the comment. To make it more precise, we have revised the sentence to “They regulate the expression of neighboring genes via specific RNA motifs, and mutant phenotype analysis implies that they might be associated with plant size and seed morphology” (page 21, line 610, marked in blue). Thank you again for all your suggestions.*

Reviewer #2:

In this work, Zhang and co-workers present the first identification, classification and partial characterization of cbromatin-associated lncRNAs, the so-called cheRNAs, in plants. To this end, they have performed RNA-seq of chromatin vs nucleoplasm fractions of previously isolated nuclei, obtained from different tissues, notable including in vitro grown calli. Based on a primary identification, the authors proceeded to a classification of novel chromatin-associated lncRNAs according to their anatomy regartding neighboring genes and including a set of correlations of transcriptional activity and potential action in cis or in trans. Finally, they characterized the physiological role of a subset of selected cheRNAs. As an interesting reader in plant lncRNAs, I find this work important and valuable for the broad audience of GB. It is very well written and it's easy to follow. Congratulations! However, I still have a few major and minor concerns that I think that should be addressed prior to publication:

Major concerns:

Comment 1. Page 5, line 42: the authors state that all the analyses are based on 2 biological raplicates. Although the main aim of the experiment was to identify transcripts, the truth is that authors conclude about enrichment of chromatin vs nucleoplasm, etc. I want to make it clear that I'm not an expert in stats behind RNA-seq, and I strongly recommend the Editor to assess this point together with experts from Genome Biology, and/or other reviewers. A third replicate might be requested.

*Reply: Considering the reviewer’s concern, we have performed another biological replicate for RNA-seq of the CPE, SNE and input samples of both embryo and callus tissues. As the revised manuscript should be submitted within six weeks, we could not collected differentiated callus and shoot (callus induced from embryo to differentiated stages, green spots, need about 45 days). We have de novo identified cheRNAs using both the three replicates and compared the newly identified cheRNAs with those cheRNAs identified previously using the two replicates. We found that about 90% of these cheRNAs are overlapped (see below Figure 5, and Table1), indicating that our method is stable and reproducible, and two biological replicates are fine for cheRNAs identification. In addition, we have also performed ChRIP assay to verify cheRNAs, which could further support the sequencing data. To keep the consistent and comparable of all the 12 samples, we preferred not to add the new sequencing data from the 6 samples into the revised manuscript, but we have uploaded these new sequencing data to the SRA database together with other datasets generated in this study. Hope that this can be understood. Thank you.*

Comment 2. When the authors characterized the physiological role of selected lncRNAs, they used T-DNA insertional mutants. This is risky, considering the proximity of the T-DNA and neighboring genes, which may alter their expression regardless the role of the cheRNAs. Ideally, RNAi approaches (considering that CRISPR-mediated point mutations would be useless for ncRNA loci) should be included to distinguish T-DNA insertions from the lncRNA activity. Otherwise, please at least discuss better potential dual or alternative effects of these mutants.

*Reply: This is a good comment indeed. As suggested, we have performed additional observation using the previously constructed RNAi transgenic plants of OsCHENAT1709. We have analyzed the callus dedifferentiation and tiller number phenotypes of the chenat1709-RNAi plants and found that they are similar with that of the T-DNA insertion mutant of OsCHENAT1709. In addition, we have also transfected calli with the RNAi vector of OsCHELIN2084 and analyzed the phenotypes. The calli transfected with chelin2084-RNAi vectors showed rapid proliferation and delayed regeneration, which is similar with that of the T-DNA insertion mutant of OsCHELIN2084 (see below Figure 6). These data have been added to the revised manuscript (page 16, lines 451-452, line 459, line 461, lines 466-469; marked in blue) and Additional file 1: Figure S5D-G, J, thank you.*

Comment 3. When classifying the identified cheRNAs, I think the authors should include the profile of epigenetic marks (including histone modifications, DNA methylation and chromatin accessibility, eg. ATAC-seq). Notably, considering that it has been suggested that in plants TEs are the main source of active lncRNAs, how many of these elements resembles or belong to TEs and what is their epigenetic profile?

*Reply: Thank you for the suggestion. Following the suggestion and the comment from the reviewer 1(comment 2), we have performed additional analysis to obtain the relationship between cheRNAs and epigenetic activities. We have collected most of the published data on DNA methylation, histone modification and DNase sequencing. The data on rice shoot and callus are available whereas no data on embryo and differentiated callus was reported. Thus, we examined the DNA status and histone marks on the neighboring genes of cheRNAs from those data on rice shoot and callus. We found that che-lncRNAs are positively correlated with the active histone marks H3K4me3 and H3K27ac modifications and chromatin accessibility, and negatively correlated with the suppressive histone mark H3K27me3 modification of their neighboring genes, whereas other histone modifications and DNA methylations are not affected by che-lncRNAs (see below Figure 7). Interestingly, most of the reported chromatin remodeling regulatory lncRNAs regulate gene expression through mediating H3K4me3, H3K427me3 and/or H3K27ac modifications in plants. These data imply that chromatin remodeling regulatory lncRNAs including che-lncRNAs are inclined to regulate target gene expression through mediating H3K27me3 and/or H3K4me3 modifications. The results have been added to the revised manuscript (page 12, lines 345-353, marked in blue) and figure 4F; Additional file 1: Figure S4C, D.*

*We have also analyzed the TE-derived cheRNAs as suggested. We have identified 1,117 (48.9 % of cheRNAs) TE-derived che-lincRNAs. Higher suppressive histone marks (H3K27me3 and H3K9me2) and lower active histone marks (H3K27ac and H3K4me3) were observed at the TE-derived che-lincRNAs loci compared with other che-lincRNAs (see below Figure 8). The results have been added to the revised manuscript (page 8, lines 220-225, marked in blue) and Additional file 1: Figure S2H; Additional file 2: Supplementary Table 2, thank you.*

Minor comments:

I'm a bit surprised that the authors avoided in their discussion all that is known about lncRNAs and protein partners in Arabidopsis (and even in rice). From our own group, we have shown the interaction of a lncRNA, APOLO, with PRC1-like components, LHP1, regulating genes in cis (Ariel et al 2014) and in trans forming DNA-RNA duplexes, also discussed here (Ariel et al 2020). I don't say this because I expect the authors to cite specifically our papers!! But together with this, there are several lncRNAs in plants known to interact with chromatin remodelers, driving histone (de)acetylation or (de)medthylation, what could be interesting to discuss in the frame of what I suggested above in point 3. I recommend to read the last part of one of our recent reviews (Fonouni-Farde et al 2021, in "non-coding RNA") to identify these key papers. I consider that a deeper discussing would help to merge the findings with the literature.

*Reply: We apologize for the insufficient discussion in the previous version. Indeed, several plant lncRNAs have been reported to interact with chromatin remodelers. We have added the discussion of these lncRNAs including APOLO and the potential regulatory mechanisms of cheRNAs in the revised manuscript (both in the Introduction section and Discussion section) as suggested (page 4, lines 93-94; and page 20, lines 576-591; marked in blue). In addition, according to your comment 3, we have found that che-lncRNAs probably regulate gene expressing through affecting H3K4me3, H3K27me3 and H3K27ac modifications, which is similar with those reported chromatin remodelers interacting lncRNAs you have mentioned above. Thus we discussed this hypothesis in detail in the revised manuscript. Thank you so much for the suggestion, we appreciate it.*

Page 10, line 42: what do the authors think cheRNAs are doing in the nucleoplasm? Are they released from chromatin just because they are too abundant under certain circumstances or this may be a hint of dual roles in cis vs in trans? Please, discuss. Among the different sub-classes of cheRNAs proposed by the authors, there are che-snRNAs. Strikingly, an old paper came to my mind about snRNAs regulating the capacity of TFs to bind chromatin (Higuchi et al 2007, BBA). Interestingly, the same TF (NFYA) binding snRNAs can also bind the PANDA lncRNA (Hung et al., 2011, Nat Gen). I think the authors should comment on the potential role of cheRNAs in the modulation of TFs and other chromatin-related proteins.

*Reply: It has been reported that the dissociation of lncRNAs from chromatin is important for their regulatory roles. For example, lncRNA A-ROD was shown to enhance DKK1 transcription at its release from chromatin[4]. In addition, lncRNAs are also reported to play a role in nuclear organelles such as MALAT1 in speckles and LoNa in nucleolus[5]. However, both chromatin associated lncRNAs and nucleoplasm lncRNAs could function in cis or in trans[6]. We have added the discussion about the subcellular localization of cheRNAs and the potential effects of cheRNAs on chromatin-related proteins in the revised manuscript( page 19, lines 560-565; page 20, lines 591-593; marked in blue). Thank you!*

Additional details: I strongly suggest the authors to give a name to each XLOC transcript they have characterized. They may be called by the same name-1, -2, -3, etc. But a name helps the community to identify who is who in the noncoding genome world!

*Reply: We agree and have renamed all the cheRNAs. The cheRNAs are now named according to the type of the cheRNAs and their number coordinating in chromosome. For example, OsCHELIN (Rice Chromatin-Enriched Long Intergenic Noncoding RNAs), OsCHENAT (Rice Chromatin-Enriched NATs), OsCHESNO (Rice Chromatin-Enriched SnoRNAs), OsCHESN (Rice Chromatin-Enriched SnRNAs), OsCHET (Rice Chromatin-Enriched tRNAs) -1, 2 and etc. We have also provided a brief explanation in the revised manuscript (page 6, lines 157-160). Thank you for the suggestion.*

Page 7, line 32: NONCODE is misspelled.

*Reply: We have revised the word (page 7, line 209, marked in blue), thank you.*

Line 42: "28.1% of lincRNAs are located within 500 bp of coding genes…" I think "of" should be replaced by "away from".

*Reply: We have used “away from” instead of “of” (page 8, lines 214-215, marked in blue), thank you.*

Line 62: what do the author mean by "traditional lncRNAs"? "Previously characterized" sounds more accurate.

*Reply: We have used “previously characterized lncRNAs” instead of “traditional lncRNAs” (page 8, lines 232-233 and line 240; page 9, line 259; marked in blue). Thank you again for all you suggestions.*

Reference

1. Schardon K, Hohl M, Graff L, Pfannstiel J, Schulze W, Stintzi A, et al. Precursor processing for plant peptide hormone maturation by subtilisin-like serine proteinases. Science. 2016; 354(6319):1594-1597.

2. Tanaka H, Onouchi H, Kondo M, Hara-Nishimura I, Nishimura M, Machida C, et al. A subtilisin-like serine protease is required for epidermal surface formation in Arabidopsis embryos and juvenile plants. Development (Cambridge, England). 2001; 128(23):4681-4689.

3. Kim B, Piao R, Lee G, Koh E, Lee Y, Woo S, et al. OsCOP1 regulates embryo development and flavonoid biosynthesis in rice (Oryza sativa L.). Theor Appl Genet. 2021; 134(8):2587-2601.

4. Ntini E, Louloupi A, Liz J, Muino JM, Marsico A, Orom UAV. Long ncRNA A-ROD activates its target gene DKK1 at its release from chromatin. Nat Commun. 2018; 9(1):1636.

5. Carlevaro-Fita J, Johnson R. Global Positioning System: Understanding Long Noncoding RNAs through Subcellular Localization. Mol Cell. 2019; 73(5):869-883.

6. Gil N, Ulitsky I. Regulation of gene expression by cis-acting long non-coding RNAs. Nat Rev Genet. 2020; 21(2):102-117.

**Second round of review**

**Reviewer 1**

Most my questions have been addressed by the authors. I have only a suggestion before publication.

The authors generated several useful mutants of cheRNAs as well as their target genes. Since there is no information about how to produce them, I suggest that they should provide details in the materials and method section or add a Supplemental figure to introduce the mutant sites by CRISPR-cas9/T-DNA and dsRNA regions by RNAi. This will be helpful for other scientists to ask for and use these important transgenic plants.

**Reviewer 2**

I appreaciate the improvement of the manuscript. This is an excellent work in the field of plant noncoding RNAs. I find it suitable for publication in a major journal. Congratulations!

**Authors Response**

**Point-by-point responses to the reviewers’ comments:**

Reviewer #1:

Most my questions have been addressed by the authors. I have only a suggestion before publication.

The authors generated several useful mutants of cheRNAs as well as their target genes. Since there is no information about how to produce them, I suggest that they should provide details in the materials and method section or add a Supplemental figure to introduce the mutant sites by CRISPR-cas9/T-DNA and dsRNA regions by RNAi. This will be helpful for other scientists to ask for and use these important transgenic plants.

*Reply: Thank you for your suggestion. We have provided details about introducing the mutant sites for cheRNAs and their target genes by CRISPR-cas9/T-DNA and dsRNA regions by RNAi in the method section (page 25, page 28, marked in blue) and also in supplemental figure 5A in the revised manuscript.*

Reviewer #2:

I appreaciate the improvement of the manuscript. This is an excellent work in the field of plant noncoding RNAs. I find it suitable for publication in a major journal. Congratulations!

*Reply: Thank you for your comments.*
